# The genome of *Dioscorea zingiberensis* sheds light on the biosynthesis, origin and evolution of the medicinally important diosgenin saponins

**DOI:** 10.1093/hr/uhac165

**Published:** 2022-07-25

**Authors:** Yi Li, Chao Tan, Zihao Li, Jingzhe Guo, Song Li, Xin Chen, Chen Wang, Xiaokang Dai, Huan Yang, Wei Song, Lixiu Hou, Jiali Xu, Ziyu Tong, Anran Xu, Xincheng Yuan, Weipeng Wang, Qingyong Yang, Lingling Chen, Zongyi Sun, Kai Wang, Bo Pan, Jianghua Chen, Yinghua Bao, Faguang Liu, Xiaoquan Qi, David R Gang, Jun Wen, Jiaru Li

**Affiliations:** State Key Laboratory of Hybrid Rice, College of Life Sciences, Wuhan University, Wuhan, 430072, China; State Key Laboratory of Hybrid Rice, College of Life Sciences, Wuhan University, Wuhan, 430072, China; State Key Laboratory of Hybrid Rice, College of Life Sciences, Wuhan University, Wuhan, 430072, China; State Key Laboratory of Hybrid Rice, College of Life Sciences, Wuhan University, Wuhan, 430072, China; State Key Laboratory of Hybrid Rice, College of Life Sciences, Wuhan University, Wuhan, 430072, China; State Key Laboratory of Hybrid Rice, College of Life Sciences, Wuhan University, Wuhan, 430072, China; State Key Laboratory of Hybrid Rice, College of Life Sciences, Wuhan University, Wuhan, 430072, China; State Key Laboratory of Hybrid Rice, College of Life Sciences, Wuhan University, Wuhan, 430072, China; State Key Laboratory of Hybrid Rice, College of Life Sciences, Wuhan University, Wuhan, 430072, China; State Key Laboratory of Hybrid Rice, College of Life Sciences, Wuhan University, Wuhan, 430072, China; State Key Laboratory of Hybrid Rice, College of Life Sciences, Wuhan University, Wuhan, 430072, China; State Key Laboratory of Hybrid Rice, College of Life Sciences, Wuhan University, Wuhan, 430072, China; State Key Laboratory of Hybrid Rice, College of Life Sciences, Wuhan University, Wuhan, 430072, China; State Key Laboratory of Hybrid Rice, College of Life Sciences, Wuhan University, Wuhan, 430072, China; State Key Laboratory of Hybrid Rice, College of Life Sciences, Wuhan University, Wuhan, 430072, China; State Key Laboratory of Hybrid Rice, College of Life Sciences, Wuhan University, Wuhan, 430072, China; Hubei Key Laboratory of Agricultural Bioinformatics, College of Informatics, Huazhong Agricultural University, Wuhan, 430070, China; College of Life Science and Technology, Guangxi University, Nanning, 530004, China; Grandomics Biosciences, Beijing 102200, China; Grandomics Biosciences, Beijing 102200, China; Center for Integrative Conservation, Xishuangbanna Tropical Botanical Garden, Chinese Academy of Sciences, Menglun, 666303, China; CAS Key Laboratory of Tropical Plant Resources and Sustainable Use, CAS Center for Excellence in Molecular Plant Sciences, Xishuangbanna Tropical Botanical Garden, Kunming, 650223, China; Henry Fok College of Biology and Agriculture, Shaoguan University, Shaoguan, 512005, China; Henry Fok College of Biology and Agriculture, Shaoguan University, Shaoguan, 512005, China; Key Laboratory of Plant Molecular Physiology, Institute of Botany, Chinese Academy of Sciences, Beijing, 100093, China; Institute of Biological Chemistry, Washington State University, Pullman, WA, 99164, USA; Department of Botany, National Museum of Natural History, Smithsonian Institution, Washington, DC, 20013-7012, USA; State Key Laboratory of Hybrid Rice, College of Life Sciences, Wuhan University, Wuhan, 430072, China

## Abstract

Diosgenin saponins isolated from *Dioscorea* species such as *D. zingiberensis* exhibit a broad spectrum of pharmacological activities. Diosgenin, the aglycone of diosgenin saponins, is an important starting material for the production of steroidal drugs. However, how plants produce diosgenin saponins and the origin and evolution of the diosgenin saponin biosynthetic pathway remain a mystery. Here we report a high-quality, 629-Mb genome of *D*. *zingiberensis* anchored on 10 chromosomes with 30 322 protein-coding genes. We reveal that diosgenin is synthesized in leaves (‘source’), then converted into diosgenin saponins, and finally transported to rhizomes (‘sink’) for storage in plants. By evaluating the distribution and evolutionary patterns of diosgenin saponins in *Dioscorea* species, we find that diosgenin saponin-containing may be an ancestral trait in *Dioscorea* and is selectively retained. The results of comparative genomic analysis indicate that tandem duplication coupled with a whole-genome duplication event provided key evolutionary resources for the diosgenin saponin biosynthetic pathway in the *D*. *zingiberensis* genome. Furthermore, comparative transcriptome and metabolite analysis among 13 *Dioscorea* species suggests that specific gene expression patterns of pathway genes promote the differential evolution of the diosgenin saponin biosynthetic pathway in *Dioscorea* species. Our study provides important insights and valuable resources for further understanding the biosynthesis, evolution, and utilization of plant specialized metabolites such as diosgenin saponins.

## Introduction

**Figure 1:**
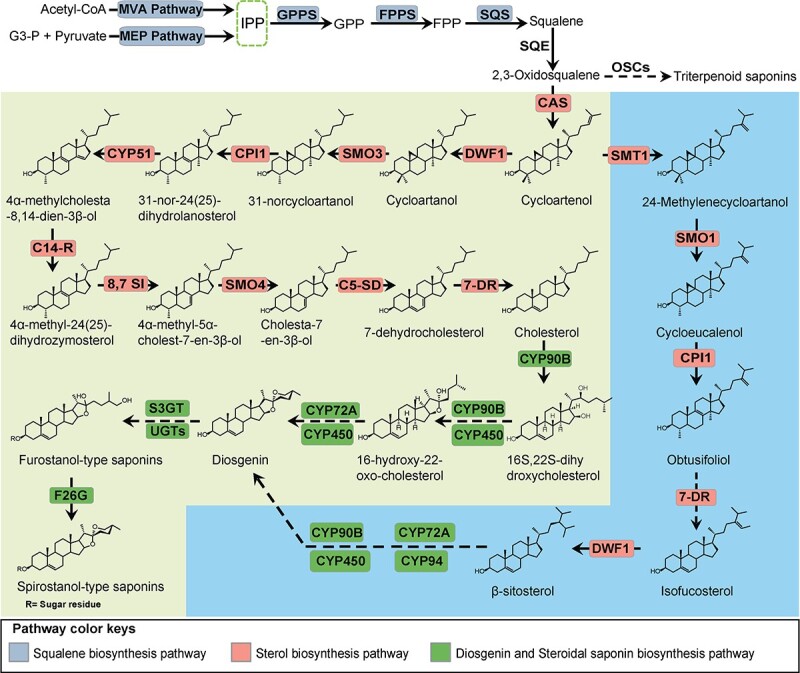
Putative biosynthetic pathway of diosgenin saponins in *D*. *zingiberensis*. The diosgenin saponin biosynthetic pathway can be divided into the following stages: synthesis of squalene, generation of cholesterol/sitosterol, biosynthesis of diosgenin, and biosynthesis of diosgenin saponins. Isopentenyl diphosphate (IPP) is synthesized through the mevalonate (MVA) and methylerythritol 4-phosphate (MEP) pathways. Three enzymes (GPPS, geranylgeranyl pyrophosphate synthase; FPPS, farnesyl diphosphate synthase; and SQS, squalene synthase) involved in the synthesis of squalene are depicted in gray. Eleven enzymes [SQE, squalene epoxidase; CAS, cycloartenol synthase; SMT, sterol C-24 methyltransferase; SMO, C-4 sterol methyl oxidase; CPI1, cyclopropylsterol isomerase; CYP51, sterol C-14 demethylase; C14-R, sterol C-14 reductase; 8,7 SI, sterol 8;7 isomerase; CD-SD, sterol C-5(6) desaturase; 7-DR, 7-dehydrocholesterol reductase; and DWF1, δ-(24)-sterol reductase] that participate in plant sterol (cholesterol and β-sitosterol) biosynthetic pathways are marked in salmon, and four key enzymes (CYP90B, sterol 22-α-hydroxylase; CYP72A/CYP94, sterol 26-α-hydroxylase; S3GT, sterol 3-β-glucosyltransferase; and F26G) involved in the synthesis of diosgenin saponins are marked in green. Dashed arrows indicate multiple steps in the pathway.

Diosgenin is a steroidal sapogenin first isolated from the rhizomes of *Dioscorea tokoro* in the 1930s [1]. Diosgenin saponins, with diosgenin acting as the aglycone backbone, are important bioactive constituents widely distributed in *Dioscorea* spp. Diosgenin saponins isolated from the rhizomes or tubers of *Dioscorea* plants are mainly divided into spirostanol-type and furostanol-type (Fig. 1), which have a broad spectrum of pharmacological activities, including antioxidative, anti-cancer, anti-inflammatory, anti-fungal, and hypolipidemic functions, and are widely used in pharmacological research and medical treatment [2–4].

The genus *Dioscorea* of the family Dioscoreaceae consists of ~600 species [5], which are mainly divided into the following lineages: *Dioscorea* sect. *Stenophora*, sect. *Combilium*, sect. *Opsophyton*, sect. *Enantiophyllum*, and New World I/II [6–8]. *Dioscorea* species are cultivated in many parts of the world not only for their synthesis of diosgenin saponins, but also as an important food crop. The tubers of some *Dioscorea* plants, such as *D. rotundata*, *D. alata* and *D. oppositifolia*, are often used as edible yams because of their high starch content, while other *Dioscorea* species, such as *D. zingiberensis*, *D. panthaica*, and *D. nipponica*, are used as medicinal plants due to their abundance of diosgenin saponins [6, 8]. More than 190 diosgenin saponins have been isolated from *Dioscorea* plants; however, only 137 species of *Dioscorea* contain diosgenin saponins [9]. Moreover, the contents and types of diosgenin saponins in these *Dioscorea* plants are different, and only 41 species contained >1% of diosgenin [9]. About 70, 30, and <10 diosgenin saponins are identified from sect. *Stenophora* species such as *D. zingiberensis*, *D. panthaica*, and *D. nipponica*, respectively [10–12]. However, in other lineages, ~15 diosgenin saponins are isolated from *D. esculenta* (sect. *Combilium*), and even fewer from *D. bulbifera* (sect. *Opsophyton*) [13, 14]. Diosgenin saponins are typically found in *Dioscorea* plants; however, why only a few species of *Dioscorea* are rich in diosgenin saponins still remains unclear.

*D. zingiberensis*, a traditional medicinal plant species rich in diosgenin saponins and diosgenin (up to 16.15% of dry weight) [15] (Supplementary Data Fig. 1), has been used in folk medicines since as early as 2000 years ago [10]. It is also used as an ideal steroid hormone source plant in the world, so it is considered an important model for investigating the biosynthesis of diosgenin saponins [16–18]. Based on previous studies, the biosynthesis of diosgenin saponins can be divided into the following stages: biosynthesis of the 30-carbon squalene via mevalonate (MVA) and methylerythritol 4-phosphate (MEP) pathways; biosynthesis of cholesterol or sitosterol as precursor; the catalysis of sterol side chains to synthesize diosgenin (cytochrome P450s, CYP450); and finally glycosylation to form diosgenin saponins (UDP-glycosyltransferases, UGTs) [19–24] (Fig. 1). Notably, the β-glucosidase furostanol glycoside 26-*O*-β-glucosidase (F26G) may be involved in the catalysis of diosgenin formation and the conversion of furostanol-type saponins to spirostanol-type saponins [20, 22] (Fig. 1). At present, the MVA, MEP, squalene, and cholesterol biosynthetic pathways are ubiquitous in plants, and these pathways are also associated with other pathways [21]. Therefore, the key to diosgenin saponin biosynthesis lies downstream of cholesterol or β-sitosterol, and this is the focus of further research. Revealing the biosynthesis of diosgenin saponins in plants will help us to understand the molecular mechanism of the formation and evolution of diosgenin saponins in plants.

In recent years, more studies have been devoted to the biosynthesis of diosgenin saponins in plants [15, 17, 24, 25], but a refined chromosome-scale reference genome of *D. zingiberensis* is still the key to deciphering the molecular mechanisms underlying this biosynthetic pathway. Here we report a high-quality chromosome-level reference genome of *D. zingiberensis*. Through comparative genomic, transcriptomic, and metabolomic analyses, we have obtained novel insights into the mechanisms driving the formation and evolution of the diosgenin saponin biosynthetic pathway. Our study lays an important foundation for further research in the molecular mechanism of diosgenin saponin biosynthesis and evolution in plants, and also provides a reference for understanding the formation and evolution of plant specialized metabolites.

**Table 1:** Statistics of genome assembly and annotation of *D. zingiberensis*.

**Genomic feature**		
Genome assembly		
	Assembled sequences (bp)	629 184 705
	Contig N50 (bp)	1 164 246
	Scaffold N50 (bp)	55 778 752
	GC content of the genome (%)	39.51
Genome annotation		
	Number of protein-coding genes	30 322
	Mean gene length (bp)	4820
	Mean coding sequence length (bp)	1215
	Mean exon length (bp)	219
	Mean intron length (bp)	792

## Results

### Genome assembly and annotation

Based on genome survey results, we estimated that the genome size of *D*. *zingiberensis* was 716 Mb with a high level of heterozygosity (1.56%) (Supplementary Data Fig. 2a); furthermore, *D*. *zingiberensis* is a diploid with a karyotype of 2*n* = 20 (Supplementary Data Fig. 2b). To overcome the difficulties in assembling the highly heterozygous genome, a combined strategy was used to produce 91.34 Gb of Illumina sequencing data (~110× coverage) for the genome survey, 117.18 Gb of Oxford Nanopore Technologies (ONT) long reads (N50 length of 19 kb, ~146×), 106.97 Gb of 10X Genomics Linked-Reads (~170×), and 194.77 Gb of Hi-C sequencing data (~310×) for genome assembly (Supplementary Data Table 1), generating a chromosome-scale genome (~629 Mb) with contig N50 of 1.16 Mb and scaffold N50 of 55.78 Mb (Table 1, Supplementary Data Table 2). Approximately 93.39% of the genomic sequences were anchored on 10 linkage groups (Supplementary Data Fig. 3, Supplementary Data Table 3). We further evaluated the quality and integrity of the assembled genome, and found that 99.30% of Illumina reads and 99.35% of Nanopore reads could be mapped to the scaffolds (Supplementary Data Table 4). In addition, Benchmarking Universal Single-Copy Orthologs (BUSCO) [26] analysis revealed that 96.84% of 1614 core eukaryotic genes were found complete in our *D. zingiberensis* genome assembly (Supplementary Data Table 5). Moreover, analysis using a Conserved Core Eukaryotic Gene Mapping approach (CEGMA) [27] showed that 97.98% of 248 conserved core eukaryotic genes from CEGMA were captured in our assembled genome (Supplementary Data Table 5). These results suggest that our genome assembly is of high quality.

**Figure 2:**
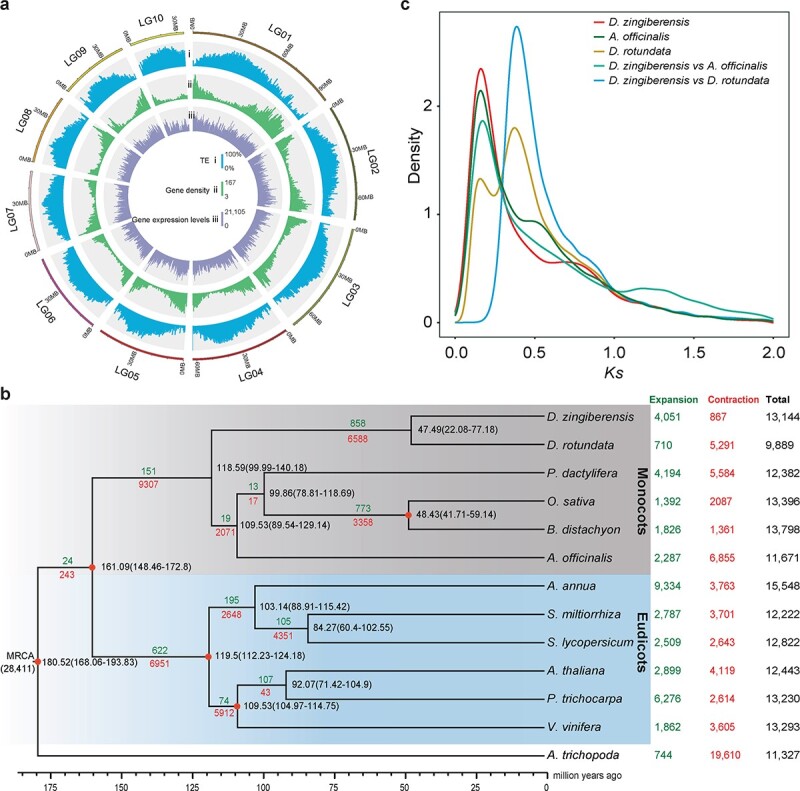
Genome assembly characterization and genome evolution of *D. zingiberensis*. **a** Circos view of the *D. zingiberensis* genome. (i) TE density distributions in each linkage group. (ii) Gene density: numbers of genes in 1-Mb non-overlapping windows. (iii) Gene expression levels: gene expression levels of each gene in leaves, stems, and rhizomes of *D. zingiberensis*. The distribution of TE density shows a peak in the middle, while that of gene density shows two peaks, with one at each end. **b** Phylogenetic tree showing the divergence times and evolution of gene family sizes for 13 plant species. The value at each node represents the divergence time in millions of years ago (Mya) relative to the present. Expansion: number of gene families expanded (green); Contraction: number of gene families contracted (red); Total: total number of gene families (black). **c** WGD events detected in *D*. *zingiberensis*. Frequency plot of *K*_s_ distributions of paralogs identified from *D*. *zingiberensis*, *D. rotundata*, and *A. officinalis*.

About 56.50% of the sequences in the *D. zingiberensis* genome were transposable elements (TEs) (Supplementary Data Table 6), and the distribution density of TEs in the genome generally increased from the distal to proximal regions of the chromosome arms, whereas gene density was reversed (Fig. 2a), which was consistent with previous results [28, 29], indicating the high accuracy of our assembly. We predicted a total of 30 322 protein-coding genes, and the mean lengths of the gene and coding sequence were 4820 and 1215 bp, respectively (Supplementary Data Fig. 4, Table 1, Supplementary Data Table 7). Over 94% of these predicted genes had functional annotation matches to the public databases (Supplementary Data Fig. 5, Supplementary Data Table 8). On average, >93% of RNA-seq reads from the leaves, stems, and rhizomes of *D*. *zingiberensis* could be mapped to the genome (Supplementary Data Table 9), reflecting the high reliability of genome assembly and annotation.

### Comparative genomic analysis

To investigate the evolutionary history of the *D. zingiberensis* genome, the *D. zingiberensis* genome was compared with those of 12 other representative plant species. In total, we identified 13 144 gene families in the *D. zingiberensis* genome by OrthoMCL [30], of which 745 were specific to *D*. *zingiberensis* (Supplementary Data Fig. 6, Supplementary Data Table 10). We found 7741 genes that were specific to *D*. *zingiberensis* compared with these 12 selected plant species (Supplementary Data Fig. 7, Supplementary Data Tables 10 and 11). Then, we examined the functions of *D*. *zingiberensis*-specific genes based on both the KEGG (Kyoto Encyclopedia of Genes and Genomes) and GO (Gene Ontogeny) databases, and found that these genes were mainly enriched in ‘secondary metabolic processes’, especially UGT activity (GO:0008194, *P* < .001) (Supplementary Data Fig. 8), potentially representing several key steps in the biosynthesis of diosgenin saponins.

We then constructed the phylogenetic relationships and estimated the divergence times of the 13 angiosperm plant species, based on the 430 single-copy orthologous genes retrieved using OrthoMCL [30] (Fig. 2b). The results suggested that *D*. *zingiberensis* is sister to *D. rotundata* with an estimated divergence time of ~47.49 million years ago (Mya). The diosgenin-containing species *Phoenix dactylifera* of Palmae [31] and *Asparagus officinalis* of Asparagaceae [32] are in a clade sister to the *Dioscorea* clade (Fig. 2b).

Whole-genome duplication (WGD) events are important in the duplication and retention of genes, especially those related to plant species-specific phenotypic traits, and are ubiquitous in terrestrial plant genomes [33–35]. Here we identified 21 257, 14 589, and 14 128 paralogous gene pairs in the *D*. *zingiberensis*, *D. rotundata*, and *A. officinalis* genome, respectively. Two peaks of synonymous substitutions per synonymous site (*K*_s_) distribution were detected for the *D. zingiberensis* genome at ~0.14 and ~ 0.74, respectively, suggesting that two WGD events occurred around 12–22 and 83–93 Mya in the evolutionary history of *D*. *zingiberensis* (Fig. 2b, Supplementary Data Fig. 9). Similarly, we found two peaks of *K*_s_ distribution of *D. rotundata*, while the second one (~0.41, ~44–54 Mya) nearly coincided with the *K*_s_ between *D. zingiberensis* and *D. rotundata* (~0.40, ~48 Mya) (Fig. 2c, Supplementary Data Fig. 9). The younger WGD event around 12–22 Mya in *D. zingiberensis* was after the divergence of *D. zingiberensis* and *D. rotundata* at ~47.49 Mya (Fig. 2b and c), indicating a species-specific WGD event in the genome of the *D*. *zingiberensis* lineage.

By comparing orthologous genes between *D*. *zingiberensis* and 12 selected plant species, we found that expanded gene families (4051 gene families, including 6873 genes) substantially outnumbered contracted gene families (867 gene families, including 1088 genes) in the *D*. *zingiberensis* genome (Fig. 2b, Supplementary Data Table 12). KEGG and GO enrichment analysis suggested that these expanded genes were mainly enriched in steroid biosynthesis (ath00100, *P* < .001) and UGT activity (GO:0008194, *P* < .001), respectively (Supplementary Data Fig. 10). The expanded gene families included *OSC* (PF13243), *CYP450* (PF00067), and *UGT* (PF00201), whose members function in several key steps in the biosynthesis of diosgenin saponins [19], suggesting an evolutionary basis for the higher capacity for diosgenin saponin biosynthesis in *D*. *zingiberensis* compared with other plant species.

### Biosynthesis of diosgenin saponins in *D. zingiberensis*

**Figure 3:**
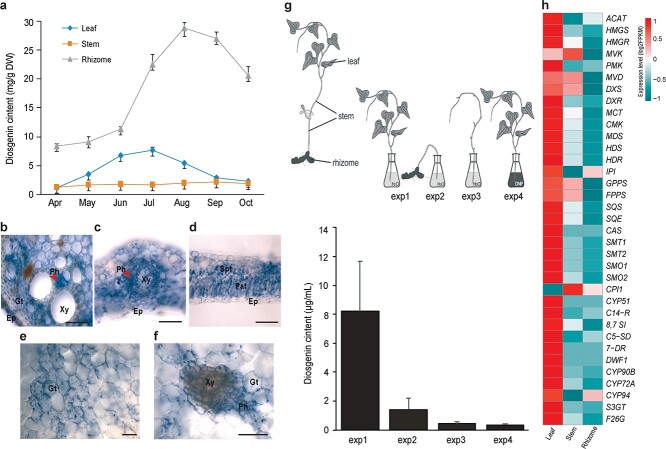
Diosgenin distributions and gene expression levels in different tissues of *D. zingiberensis*. **a** Diosgenin contents in different tissues of *D*. *zingiberensis* during the development period of 7 months. **b**–**f** Immunohistochemical localization of diosgenin in stem (**b**), leaf (**c**, **d**), and rhizome (**e**, **f**) tissues. Blue color in sections represents the diosgenin signal. Diosgenin is detected in the epidermis (Ep), ground tissue (Gt), and phloem (Ph) of the stem cross-section. Blue signals localized in the phloem are observed. The diosgenin signal is concentrated in palisade tissue (Pat), spongy tissue (Spt), and phloem in the leaf. Xy represents the xylem. Scale bar = 100 μm. **g** Schematic diagram of the transport assay. The aerial part of the plant with leaves attached (exp1), stem connected to the rhizome (exp2), and stem of the aerial part with leaves detached were immersed in flasks containing deionized water (exp3). The aerial part with leaves attached was also inserted into a flask containing 1.5 mM dinitrophenol (exp4). The diosgenin content secreted into the water in each treatment is shown in the histogram. **h** Expression profiles of pathway genes based on RNA-seq data from three tissues (leaves, stems, and rhizomes). Gene expression values (FPKM) were normalized by log2, and the expression pattern was visualized using the R package pheatmap. Three biological replicates for each sample were collected in this study, and the error bar represents the standard error.

In order to fully understand the spatial and temporal change of diosgenin saponins in *D. zingiberensis*, we measured the contents of diosgenin and diosgenin saponins in three different tissues (leaves, stems, and rhizomes) of *D*. *zingiberensis*. The diosgenin content first peaked in leaves in July, and then in rhizomes in August. The content of diosgenin in stems was always lower than that in leaves and rhizomes (Fig. 3a).

Histological localization of diosgenin was determined using a rabbit polyclonal anti-diosgenin antibody that we developed previously [36]. Strong diosgenin signals were found in the phloem of vascular tissues in the leaf vein, stem, and rhizome (Fig. 3b, c, and f). Diosgenin signals were also observed in the palisade and spongy parenchyma tissues of the leaf (Fig. 3d). In the rhizome, the diosgenin signal appeared as condensed particles scattered inside the parenchyma cells (Fig. 3e and f), and control slides are shown in Supplementary Data Fig. 11. Diosgenin signals were also observed in the epidermis and ground tissues of the stem. As the phloem is mainly responsible for the downward transport of organic compounds, our results suggest that phloem in vascular tissues may play a role in diosgenin transport, while leaves and rhizomes act as sites of its biosynthesis and accumulation, respectively.

To test this hypothesized transport route, we measured diosgenin content in the exudate obtained from cut sites in dissected plant tissues (Fig. 3g). The stem connected to the rhizome secreted a negligible amount of diosgenin (1.41 μg/ml). However, the diosgenin content obtained from the aerial parts was substantially higher (8.22 μg/ml, Fig. 3g), suggesting that diosgenin may be transported in a downward manner. On the other hand, the aerial part with all leaves removed secreted as little as 0.42 μg/ml diosgenin (Fig. 3g). The transport of diosgenin was investigated further by immersing the aerial part of the plant in 1.5 mM 2,4-dinitrophenol solution to inhibit active transport via phloem in the stem, and the diosgenin content measured under this condition was only 0.34 μg/ml (Fig. 3g). These results support the crucial function of phloem in diosgenin transport, and also indicate that the phloem, rather than xylem, in the stem is responsible for the transport of diosgenin in *D*. *zingiberensis.*

The possibility that the rhizome was capable of diosgenin synthesis as well as storage was also investigated. We focused on the upstream genes (*SQE*, *CAS*) and downstream gene (*F26G*) of the diosgenin saponin biosynthetic pathway. Based on the expression profiles of genes analyzed by real-time qPCR, we found a trend in the gene expression levels of *SQE* and *CAS* in leaves, stems, and rhizomes: the expression of these two genes in leaves first peaked in July, but peaked in August in stems and rhizomes (Supplementary Data Fig. 12a and b). In contrast, the expression of *F26G* was also highest in leaves in July, but highest in August and September in stems and rhizomes, respectively (Supplementary Data Fig. 12c). The variation trend of total diosgenin saponins in the three tissues of *D*. *zingiberensis* was the same as that of diosgenin (Supplementary Data Fig. 12d). With the aid of our assembled genome sequences for *D*. *zingiberensis*, transcriptomic data were used to analyze the expression levels of 167 genes involved in diosgenin saponin biosynthesis in the leaves, stems, and rhizomes of *D*. *zingiberensis* (Supplementary Data Table 13). Our results showed that the highest expression levels of most of these pathway genes were detected in leaves, while the lowest levels were detected in rhizomes (Fig. 3h).

In summary, our above results indicate that diosgenin is first synthesized in leaves (‘source’), then converted into diosgenin saponins, and finally transported to underground rhizomes (‘sink’) for storage, while the leaves are essential for the synthesis of diosgenin saponins.

### Origin of diosgenin saponins in *Dioscorea* species

**Figure 4:**
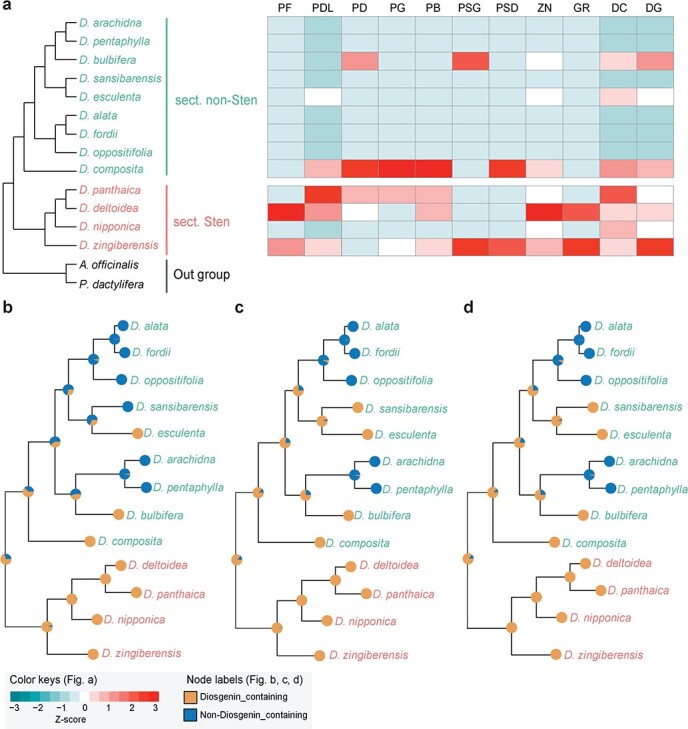
Distribution of diosgenin saponins and reconstruction of ancestral states in 13 *Dioscorea* species. **a** Left panel represents the phylogenetic relationship of the 13 *Dioscorea* species; plants from sect. *Stenophora* (sect. Sten) and non-*Stenophora* (sect. non-Sten) are represented by salmon and cyan, respectively. The content of diosgenin and diosgenin saponin detected in the leaves of each *Dioscorea* plant is displayed in the right panel. The content values are normalized using the *z*-score method. **b**–**d** Ancestral state reconstructions of three chemical traits: (**b**) furostanol-type saponins, (**c**) spirostanol-type saponins, and (**d**) diosgenin (DG). The maximum likelihood tree was constructed from the transcriptome data of 13 *Dioscorea* species and ancestral states were estimated using the R package phytools. The pie charts at the tips and nodes indicate the states of each *Dioscorea* species and the probability that ancestors contained diosgenin saponins (brown) or no diosgenin (blue).

According to the chemical structure of aglycone backbone, diosgenin saponins can be classified into two types, spirostanol-type and furostanol-type [19, 22]. In order to uncover the origin and evolution of the diosgenin saponin biosynthetic pathway, we first measured seven furostanol-type saponins (PF, parvifloside; PD, protodioscin; PB, protobioside; PG, protogracillin; PSG, pseudoprotogracillin; PSD, pseudoprotodioscin; PDL, protodeltonin), three spirostanol-type saponins (DC, dioscin; GR, gracillin; ZN, zingiberensis newsaponins), and diosgenin (DG) in leaf samples of 13 *Dioscorea* species by liquid chromatography–mass spectrometry (LC–MS) (Fig. 4a, Supplementary Data Tables 14 and 15). The average content of spirostanol-type saponins in the species of *Dioscorea* sect. *Stenophora* was 1.63 mg/g, significantly higher than that in non-*Stenophora* species (~3.4-fold on average, *P* < .05). Similarly, furostanol-type saponins varied in sect. *Stenophora* plants, but the average content of 4.7 mg/g was 3-fold more than that of non-*Stenophora* species (1.52 mg/g, *P* < .05) (Supplementary Data Table 15). The average content of DG in sect. *Stenophora* was 0.31 mg/g, which was ~3.9-fold higher than that in non-*Stenophora* samples (*P* < .05) (Supplementary Data Table 15). It is worth noting that diosgenin saponin contents are also high in *D. composita*, which belongs to the earliest diverging *Dioscorea* clade (New World I) [8] (Fig. 4a). The content of diosgenin saponins in *D. composita* can account for 3.68% of dry weight, and the yield of *D. composita* tubers is 45–90 tons per hectare, which is also a potential plant source of steroid drugs [37]. Our results suggest that diosgenin saponins are mainly distributed in sect. *Stenophora* of the *Dioscorea* genus, and *D. zingiberensis* contains all 10 types of diosgenin saponins, but *D. bulbifera*, *D. composita*, and *D. esculenta* in non-*Stenophora*, only contain certain types of diosgenin saponins (Fig. 4a, Supplementary Data Table 15).

To investigate the evolution and distribution pattern of diosgenin saponin in *Dioscorea* species (Fig. 4a), the ancestral states of binary traits (presence/absence of diosgenin saponins) were reconstructed based on the maximum likelihood tree of the transcriptome data of these 13 *Dioscorea* species. Several strong phylogenetic signals were detected in furostanol-type saponins such as PF, PB, PSG, and PSD (*P* < .05), and the estimated *λ* values were close to 1 (0.999), indicating that furostanol-type saponins in *Dioscorea* species were as expected under the Brownian motion model (Fig. 4b, Supplementary Data Table 16). The phylogenetic signals of most diosgenin saponins (furostanol-type and spirostanol-type) were mainly randomly distributed in the *Stenophora* clade, while those of DC and DG were also distributed in the non-*Stenophora* clade, such as *D. composita*, *D. esculenta*, and *D. sansibarensis* (Fig. 4c and d**,**Supplementary Data Table 16).

**Figure 5:**
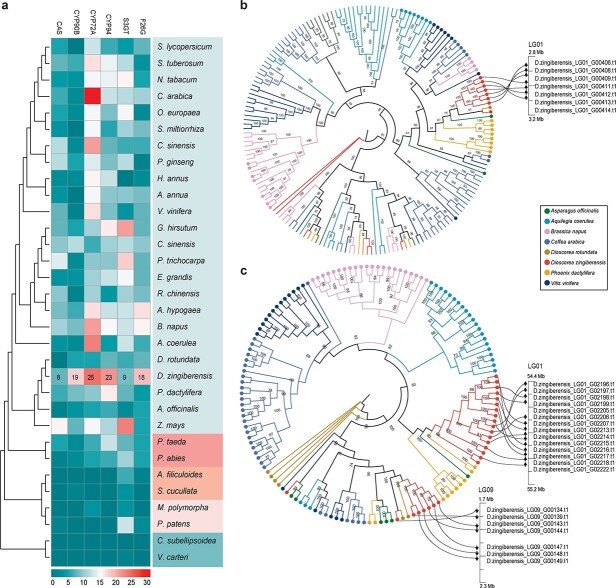
Expansion of *OSC* and *CYP72A* gene families in *D. zingiberensis*. **a** Heat map of copy number of the six key genes (*CAS*, *CYP90B*, *CYP72A*, *CYP94*, *S3GT*, *F26G*) in 32 representative plant species. The right panels show angiosperms, gymnosperms, algae, pteridophytes, and bryophytes from top to bottom. The color in each block shows the copy number of these six key genes identified in each plant. **b**, **c** Phylogenetic trees show *OSC* (**b**) and *CYP72A* (**c**) gene families in *D. zingiberensis* and seven other plant species (*Aquiligia coerulea*, *A. officinalis*, *Brassica napus*, *Coffea arabica*, *D. rotundata*, *P. dactylifera*, and *Vitis vinifera*). The dots at the end of the branches in (**b**) represent the *CAS* genes in the OSC gene family of each species. The dots at the end of the branches in (**c**) represent the *CYP72A* genes of each species. The tandem-duplicated genes are shown on the chromosomes of the *D. zingiberensis* genome.

In addition, we reconstructed the ancestral state of diosgenin saponin on a larger species scale using the collected binary diosgenin saponin phenotypes of 91 *Dioscorea* species. Our results showed that a significant phylogenetic signal was detected in *Dioscorea* species (*λ* = 0.7659, *P* < .001), and diosgenin saponin was mainly distributed in the sect. *Stenophora* species, the early diverged lineage in *Dioscorea* [8], and was scattered in non-*Stenophora* species (Supplementary Data Fig. 13). These results indicated that diosgenin saponins were mainly distributed in the early diverged lineages of *Dioscorea*, and their distribution in *Dioscorea* showed phylogenetic conservation. Furthermore, almost all evaluated ancestral nodes were located at the base of the species tree, further indicating that diosgenin saponin-containing may be an ancestral trait in *Dioscorea*.

### Evolution of the diosgenin saponin biosynthetic pathway

Based on the results of comparative genomic analysis, we found a large number of expanded gene families in *D. zingiberensis* (Fig. 2b). Considering the key roles of *OSC*, *CYP90B*, *CYP72A*, *CYP94*, *S3GT*, and *F26G* in diosgenin saponin biosynthesis [19–24], we identified these six genes in *D. zingiberensis* and 93 other representative plants, including angiosperms, gymnosperms, pteridophytes, bryophytes, and algae. The results showed that the *CYP90B*, *CYP72A*, and *CYP94* gene families were expanded in *D. zingiberensis* compared with 93 other plants, and the *F26G* gene had the most copies (Fig. 5a, Supplementary Data Table 17). Not only that, the *CYP90B*, *CYP72A*, and *CYP94* gene families in *D. zingiberensis* accounted for a higher proportion of the CYP450 gene family than in other plants (Supplementary Data Table 17).

In plants, the *OSC* gene family is involved in sterol and triterpene biosynthesis, and the *CAS* genes are responsible for sterols and diosgenin saponin biosynthesis [19, 38]. Although there were only 14 *OSC* copies in the *D. zingiberensis* genome, *D. zingiberensis* had a higher proportion of *CAS* genes (8 of 14) than the other plant species (Supplementary Data Table 17). According to the phylogenetic tree, the *CAS* genes were grouped into single clade (Fig. 5b). In contrast, compared with other plants, *D. zingiberensis* had both the highest copy number and the highest proportion of *CYP72A* in the *CYP450* gene family (Fig. 5b, Supplementary Data Table 17), and the *CYP72A* genes were mainly clustered into two clades (Fig. 5c). The phylogenomic analyses indicated that the *CAS* and *CYP72A* genes are duplicated in *D. zingiberensis*.

When we counted the number of gene copies produced by tandem duplication according to the gene locations, we found that 7 out of 8 *CAS*, 13 out of 19 *CYP90B*, 21 out of 25 *CYP72A*, 6 out of 23 *CYP94*, and 13 out of 18 *F26G* genes were produced by tandem duplication (Supplementary Data Fig. 14, Supplementary Data Table 18). Our results suggested that tandem duplication acted as an evolutionary driver for the expansion of pathway gene families. Further phylogenetic analysis showed that the *CYP90B*, *CYP72A*, *CYP94*, and *F26G* genes from *Dioscorea* species, and two diosgenin saponin-containing species (*P. dactylifera* and *A. officinalis*) were more similar (Supplementary Data Fig. 15).

Furthermore, based on the *K*_s_ value of each gene pair in these 13 *Dioscorea* species, we evaluated the gene duplication time and the contribution of paralogous duplication to the evolution of diosgenin saponin biosynthetic pathways. The results showed that only a few diosgenin saponin biosynthesis genes from diosgenin-containing *Dioscorea* species duplicated when the WGD event occurred, while the majority of the pathway genes duplicated more recently (~1–10 Mya) (Supplementary Data Fig. 16, Supplementary Data Table 19). In contrast, we found that, unlike other *Dioscorea* species, most of the diosgenin saponin biosynthetic pathway genes in *D. zingiberensis*, especially *CAS*, *CYP90B*, *CYP94*, *CYP72A*, *S3GT*, and *F26G*, were duplicated via the WGD event that occurred ~12–22 Mya, while most *CYP94* seemed to be duplicated earlier (Supplementary Data Fig. 9, Supplementary Data Table 19). A total of 8 *CAS*, 19 *CYP90B*, 23 *CYP94*, 22 *CYP72A*, 7 *S3GT*, and 18 *F26G* genes were found in the *D*. *zingiberensis* genome, of which 4 *CAS*, 4 *CYP90B*, 2 *CYP94*, 6 *CYP72A*, 2 *S3GT*, and 6 *F26G* genes were generated through the WGD event (Supplementary Data Fig. 9, Supplementary Data Table 19). Our findings indicate that tandem duplication and the WGD event may be the driving force for the evolution of the diosgenin saponin biosynthetic pathway.

### Expression pattern of the key genes involved in diosgenin saponin biosynthesis

**Figure 6:**
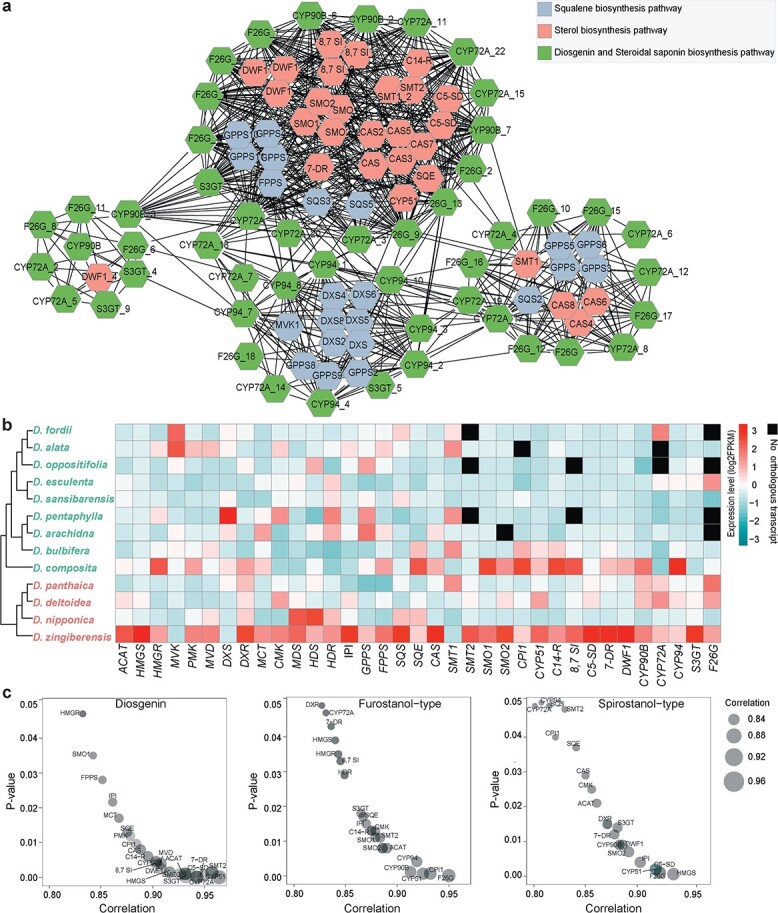
Relationships between metabolites and gene expression levels. **a** Co-expression network of diosgenin saponin biosynthetic pathway genes. Pathway genes with a Pearson correlation coefficient >.9 (*P* ≤ .01) are displayed on the network. Genes marked in gray, green, and salmon represent genes in the pathways of squalene synthesis, sterol synthesis, and diosgenin and diosgenin saponin synthesis, respectively. **b** Expression profiles of genes involved in diosgenin saponin biosynthesis in 13 *Dioscorea* species, with pathway genes shown in columns. The left panel is the phylogeny of 13 *Dioscorea* species. Expression data are plotted as log2 values. **c** Correlation analysis of gene expression with the contents of diosgenin, furostanol-type saponins, and spirostanol-type saponins. The horizontal axis of the bubble plot shows the Pearson’s correlation coefficients, and the vertical axis represents the *P* value. Circle sizes correspond to the value of Pearson’s correlation coefficient. The significance thresholds of correlations among the pathway genes are set at Pearson’s correlation coefficient value >.8 and *P*-value <.05.

Genes involved in specific metabolic pathways usually show a lineage-specific pattern of gene expression or co-expression within the transcriptome [39, 40]. Given that genes in the same metabolic pathway are often co-expressed with each other [40], we used transcriptome data of *D. zingiberensis* to explore whether there is co-expression among all genes involved in diosgenin saponin biosynthesis. The results of co-expression analysis indicated that there is indeed co-expression between genes upstream and downstream of cholesterol. Specifically, we found that *CYP94* genes were highly co-expressed with *MVK*, *DXS*, and *GPPS* genes, and *CYP72A*, *CYP90B*, and *F26G* genes were highly co-expressed with *GPPS*, *SQS*, *SMT*, *SMO*, and *CAS* genes (Fig. 6a). In particular, almost all *CAS* genes were co-expressed with *CYP90B*, *CYP72A*, and *F26G* genes; interestingly, most *CAS* and *CYP72A* genes were located on chromosome 1. At the same time, there was also co-expression among the downstream genes, such as *CYP90B*, *CYP72A*, *CYP94*, *S3GT*, and *F26G* (Fig. 6a).

We further analyzed the association between the expression of diosgenin saponin biosynthetic pathway genes in 13 *Dioscorea* plants and their diosgenin saponin content. The expression levels of almost all diosgenin saponin synthesis pathway genes, especially *CAS*, *SMO*, *CPI1*, *C14-R*, *8,7 SI*, *C5-SD*, *7-DR*, *DWF1*, *CYP94*, and *S3GT*, were highly expressed in *D*. *zingiberensis*, and *D. composita* (Fig. 6b). It is worth noting that these nine genes were expressed at a low level in *Dioscorea* species that lacked diosgenin saponins, and that no expression of *SMT2*, *CPI1*, *8,7 SI*, *CYP72A*, and *F26G* or their homologs was detected in these latter species (Fig. 4a and b). In fact, transcriptome and metabolite correlation analysis showed that the expression patterns of *CAS*, *CYP90B*, *CYP72A*, *CYP94*, *S3GT*, and *F26G* in these 13 *Dioscorea* plants was significantly correlated with the content of diosgenin saponins (Pearson’s correlation coefficient >0.9, *P* < .05) (Fig. 6b and c).

Combined with the results of gene co-expression analysis, these key genes, such as *CYP90B*, *CYP72A*, *CYP94*, *S3GT*, and *F26G*, are potentially involved in the biosynthesis of diosgenin saponins. Moreover, the expression patterns of genes involved in diosgenin saponin biosynthesis correlate well with phenotypic differentiation of diosgenin saponins in different *Dioscorea* species.

## Discussion

In this study, we report a high-quality reference genome sequence of *D*. *zingiberensis* at the chromosome level, providing the genetic basis for studying the biosynthesis and evolution of diosgenin saponins, a group of specialized metabolites of great medicinal and economic value. Our genomic database of *D*. *zingiberensis* at the chromosomal scale is particularly important for enhancing the breeding improvement of the diosgenin saponin-containing *Dioscorea* species.

Although recent transcriptome analyses of several diosgenin-containing plants, including *D. composita*, *D*. *zingiberensis*, *Trillium govanianum*, and *Trigonella foenum-graecum*, have provided insights into key genes involved in diosgenin saponin biosynthesis [37, 41–43], the biosynthetic sites of diosgenin saponins in plants remain unclear. Integrating genomic and transcriptomic data and immunohistochemical localization, our results suggested that although the rhizomes of *D. zingiberensis* are used as the medicinal parts of the plant due to being the site of accumulation, diosgenin saponins are synthesized in the leaves, and then transported through the stem vascular tissues to the underground rhizomes for storage (Fig. 3). Therefore, the leaves play a decisive role in diosgenin saponin biosynthesis in plants.

By reconstructing the ancestral state of diosgenin saponins in *Dioscorea* species, we evaluated ancestral nodes at the base of the *Dioscorea* species tree (Fig. 4b–d, Supplementary Data Fig. 13). In addition, the results of a phylogenetic signal test also showed that the distribution of diosgenin saponins in *Dioscorea* is phylogenetically conserved or phylogenetically clustered (Supplementary Data Fig. 13, Supplementary Data Table 16). All these results indicate that diosgenin saponin-containing may be an ancestral trait in *Dioscorea* species and originates from the early diverged lineages of *Dioscorea* species, sect. *Stenophora* [8]. This chemical trait (diosgenin saponin) might have formed long ago, but gradually disappeared in many *Dioscorea* species during evolution. For example, a few *Dioscorea* species, such as *D. alata*, *D. oppositifolia*, and *D. fordii*, have been artificially selected and domesticated as edible yams and appear to have lost their ability to synthesize diosgenin saponins.

The expansion or contraction of gene families is an important factor driving species differentiation, phenotypic diversification, and adaptation based on natural variation [44, 45]. Similarly, WGD events are also considered to be the major driving force in species diversification and trait variation [33–35]. In the *D. zingiberensis* genome, we found that >70% of the key genes for diosgenin saponin biosynthesis (*CAS*, *CYP90B*, *CYP72A*, and *F26G*) were replicated by tandem duplication and WGD (Fig. 5, Supplementary Data Fig. 14, Supplementary Data Tables 18 and 19). Gene duplication can help plants acquire more gene copies, which may lead to new gene functions that are more adaptable than those of single-copy or low-copy genes [35], and this also applies to *D. zingiberensis* and other *Dioscorea* plants containing diosgenin saponins. For example, sterol side-chain catalytic genes (such as *CYP90B*, *CYP94*, and *CYP72A*) played important roles in the synthesis of diosgenin saponins [25, 46, 47]. We found that the *CYP90B* genes in plants without diosgenin saponins were involved in catalyzing the conversion of cholesterol to 22S-hydroxycholesterol, en route to brassinosteroid hormones, while in plants with diosgenin saponins, such as *Dioscorea* and *Paris*, some *CYP90B* genes produced by duplication evolved sterol 16,22-polyhydroxylase activity, leading to the biosynthesis of the core structure of diosgenin [24, 25]. Although some studies have shown that *CYP72A* genes in plants are usually involved in plant-specific metabolism and the 13-hydroxylation of gibberellins in plants [48, 49], *CYP72A* genes identified from diosgenin saponin-containing plants have been shown to be involved in the biosynthesis of diosgenin saponins [23, 24, 47].

Furthermore, the WGD event can also provide genetic resources for the formation and evolution of plant-specific metabolite biosynthetic pathways, which have been revealed in some plants, such as tea, yew, and *Scutellaria baicalensis* [50–52]. Therefore, the expansion of *CAS*, *CYP90B*, *CYP94*, *CYP72A*, *F26G*, and *S3GT* genes by tandem duplication and a WGD event provided genetic and evolutionary resources for the core modification of diosgenin saponins, and also provided opportunities for the formation of the diosgenin saponin biosynthetic pathway in *D. zingiberensis*.

Gene expression patterns were also associated with phenotypic differences of diosgenin saponins in plants, which was confirmed in the correlation analysis of gene expression and diosgenin saponin content (Fig. 6c). Specifically, we detected high contents of diosgenin saponins in sect. *Stenophora* of the *Dioscorea* genus, such as *D. panthaica*, *D. deltoidea*, *D. nipponica*, and *D. zingiberensis*, and found high expression levels of genes related to diosgenin saponin biosynthesis in these plants. In *D. sansibarensis*, only a small amount of diosgenin saponins were detected, and its *CYP90B*, *CYP72A*, and *F26G* genes were also expressed at low levels (Fig. 6b). Moreover, we found that there was co-expression between the genes upstream and downstream of cholesterol identified in *D. zingiberensis* (Fig. 6a). Our results indicate that genes involved in diosgenin saponin biosynthesis often exhibit lineage-specific patterns of gene expression or co-expression, which is an important mechanism of specialized metabolism evolution [40].

In summary, our findings suggest that tandem duplications and a WGD event may be the driving forces for the species-specific evolution of diosgenin saponin biosynthesis in *D*. *zingiberensis*. Through tandem duplication and a WGD event, an increasing number of genes were produced in plants to modify the diosgenin precursor cholesterol, ultimately resulting in diosgenin saponins, and the species-specific expression patterns of pathway genes promote phenotypic differentiation of diosgenin saponins in *Dioscorea* species. Our study provides a high-quality genome of *D*. *zingiberensis*, and sets an important foundation for future research on the molecular mechanism of diosgenin saponin biosynthesis and evolution in plants.

## Materials and methods

### Plant materials

For genome sequencing, seeds of *D. zingiberensis* were collected from Shiyan City, Hubei Province, China, and cultivated in the greenhouse of Wuhan University in October 2015. Fresh tender leaves, stems, and rhizomes of *D. zingiberensis* were harvested in July 2018. These samples were immediately frozen in liquid nitrogen after collection, followed by preservation at −80°C in the laboratory prior to genome sequencing, transcriptome sequencing, and metabolite analysis.

In addition, we collected 12 *Dioscorea* plant species, among which *D. sansibarensis*, *D. composita*, *D. alata*, and *D. bulbifera* were collected from Xishuangbanna, Yunnan Province; *D. deltoidea* and *D. panthaica* were collected from Lijiang, Yunnan Province. *D. pentaphylla*, *D. esculenta*, and *D. fordii* were collected from Shaoguan, Guangdong Province; *D. oppositifolia* was collected from Xinxiang, Henan province; *D. arachidna* was collected from Wenshan, Yunnan Province; *D. zingiberensis* was collected from Xunyang, Shaanxi Province; and *D. nipponica* was collected from Fushun, Liaoning Province (Supplementary Data Table 14). These collected *Dioscorea* plants included seven lineages: sect. *Stenophora*, sect. *Enantiophyllum*, sect. *Lasiophyton*, sect. *Opsophyton*, sect. *Combilium*, New World I clade, and the Malagasy clade. We planted these *Dioscorea* plants in the greenhouse of Wuhan University, and collected the youngest leaves. All the leaf samples for transcriptome sequencing and metabolite analysis comprised three and six biological replicates, respectively.

### Genome sequencing

We adopted a combination of three sequencing technologies, including ONT, 10X Genomics, Hi-C Technologies, and other Illumina second-generation sequencing platforms, for genome sequencing, survey, and assembly. Before genome sequencing, we conducted a genome survey. Briefly, we extracted genomic DNA from *D. zingiberensis* leaves and broke it into ~350 bp fragments. Subsequently, the Illumina libraries were constructed and sequenced on the Illumina Novaseq platform. High-molecular-weight genomic DNA was extracted from tender leaves of *D*. *zingiberensis* with the Qiagen Genomic DNA extraction kit. For ONT sequencing, high-quality genomic DNA libraries were loaded into Nanopore Grid ION X5 sequencer (Oxford Nanopore Technologies) flow cells for single-molecule real-time sequencing. The 10X Genomics libraries were prepared from genomic DNA, and Illumina Hiseq XTen was used for sequencing. The Hi-C libraries were prepared using an optimized protocol [53], and then quantified and sequenced using the Illumina Hiseq platform.

### Genome assembly

To estimate the genome size and heterozygosity of *D*. *zingiberensis*, we performed *K*-mer analysis using KMC [54] and GenomeScope [55] with 350 bp of Illumina paired-end reads. First, we analyzed the *K*-mer frequency distribution. Then, we calculated the genome size based on the formula: *G* = *K*-mer number/*K*-mer depth, where *G* is genome size, the *K*-mer number is the total number of *K*-mers (*K* = 17), and *K*-mer depth is estimated from the *K*-mer distribution.

All ONT sequencing data were corrected using Nextdenovo (https://github.com/Nextomics/NextDenovo). Then, SMARTdenovo (https://github.com/ruanjue/smartdenovo) was used for genome assembly. To further improve the accuracy of genome assembly, genome correction was performed using Illumina reads mapped using minimap2 [56] and NextPolish [57]. Then, 10X Genomics Linked-Reads were used to refine assembly using ARKS and LINKS [58, 59]. Finally, we combined the filtered Hi-C data and used the *de novo* assembly pipeline in LACHESIS [60] software to produce chromosome groups. The completeness of the genome assembly was evaluated using BUSCO [26], and CEGMA [27].

### Genome annotation

We predicted and identified protein-coding genes using a combined strategy that incorporated repeat sequence annotation, gene structure prediction, and gene function annotation. For repeat sequence annotation, LTR_FINDER [61], MITE-Hunter [62], and RepeatMasker (http://www.repeatmasker.org/) were used to predict repeat sequences in the *D*. *zingiberensis* genome. Gene structure prediction was based on *ab initio* gene prediction and homologous protein prediction, which were performed using Augustus [63] and GeMoMa [64] software, respectively. Then, PASA [65] and TransDecoder (http://transdecoder.github.io/) were used to predict unigene sequences based on the transcriptome data. Finally, all of these predicted gene models were integrated and functionally annotated by mapping to KEGG (https://www.genome.jp/kegg/), KOG (http://www.ncbi.nlm.nih.gov/COG/), Swiss-Prot (http://www.gpmaw.com/html/swiss-prot.html), and NCBI NR (http://www.ncbi.nlm.nih.gov/RefSeq/) databases, We also used InterProScan methods (https://www.ebi.ac.uk/interpro/about/interproscan/) to predict the conserved sequences and structure domain against the GO database (http://geneontology.org/).

### Comparative genomic analyses

We selected five sequenced monocots (*A. officinalis*, *Brachypodium distachyon*, *D. rotundata*, *Oryza sativa*, and *P. dactylifera*) and six sequenced eudicots (*Arabidopsis thaliana*, *Artemisia annua*, *Populus trichocarpa*, *Solanum lycopersicum*, *Salvia miltiorrhiza*, and *Vitis vinifera*) for comparative genomic analysis, with *Amborella trichopoda* as outgroup. These plants include three monocots that can synthesize diosgenin saponins: *D. rotundata*, *P. dactylifera*, and *A. officinalis*. The eudicots comprised *S. lycopersicum*, which can also use cholesterol as a precursor to synthesize steroidal glycoalkaloids [19], and the two medicinal plants *S. miltiorrhiza*, *A. annua*. We used OrthoMCL [30] software to identify single-copy genes and ortholog genes in each species. Phylogenetic relationships between *D. zingiberensis* and the 12 other plant species were constructed by using RAxML [66] based on 430 single-copy orthologous genes identified. Then, the divergence times among these 13 plant species were estimated using the MCMCTREE program [67]. Finally, CAFÉ [68] was used to predict gene family expansion (gain) or contraction (loss) in these 13 plants. Meanwhile, we performed gene functional enrichment analysis of specific and expanded gene families in *D*. *zingiberensis* using the KEGG and GO databases.

### Genome evolution

To investigate the WGD events in the *D. zingiberensis* genome, we used the FASTKs pipeline (https://github.com/mrmckain/FASTKs) to estimate the synonymous substitution rate of the paralogous gene pairs in *D. zingiberensis*, *D. rotundata*, and *A. officinalis*. The protein sequences of these three plants were aligned using BLASTN with E-value <1e^−40^. For each BLAST pair, protein sequences were aligned using MUSCLE [69] and converted to codon-based alignments using PAL2NAL [70]. The *K*_s_ and the non-synonymous substitution rate (*K*_a_) for paralogous gene pairs in the genomes of *D. zingiberensis*, *D. rotundata*, and *A. officinalis* were calculated using PAML [67]. The *K*_s_ distribution plots were then drawn by R packages ggplot2 (https://cran.r-project.org/web/packages/ggplot2/index.html) and Mclust [71].

### Immunohistochemical localization and transport assay of diosgenin in *D. zingiberensis*

To investigate the spatial and temporal changes of diosgenin content in *D*. *zingiberensis*, we used a previously developed rabbit polyclonal antibody specific for diosgenin to determine the synthetic sites of diosgenin in leaves, stems, and rhizomes by immunochemical tissue localization [36]. For the transport assay of diosgenin in plants, the aerial part of the plant with leaves attached, the stem connected to the rhizome, and the stem of the aerial part with leaves detached were immersed in flasks containing deionized water. In addition, we used 2,4-dinitrophenol to inhibit active transport within the phloem. Finally, the amount of diosgenin in the solution was measured as described previously [36]. In addition, expression profiles of *SQE*, *CAS*, and *F26G* in various tissues of *D*. *zingiberensis* were obtained using qRT–PCR. The above experiments used three biological replicates.

### Gene expression analyses

We mapped all clean RNA-seq reads of leaves, stems, and rhizomes of *D*. *zingiberensis* to our assembled reference genome of *D. zingiberensis* by using STAR [72]. Then, we used HTSeq [73] to obtain the read numbers mapped to each gene. The number of fragments per kilobase of exon per million fragments mapped (FPKM) of each gene was calculated using RNS-Seq by Expectation Maximization (RESM; http://deweylab.github.io/RSEM/). The diosgenin saponin biosynthetic pathway genes were identified using BLASTP. The highest expressed genes in three *D*. *zingiberensis* tissues were selected to be visualized using the R package pheatmap (https://cran.r-project.org/web/packages/pheatmap/index.html).

In addition, we also collected RNA-seq data of three tissues from our study and existing transcriptome data of *D*. *zingiberensis* [43] to construct the co-expression network of all diosgenin saponin biosynthetic pathway genes by the Pearson correlation coefficient method in R software. We screened genes with correlation coefficient >.9 and then used these genes to construct gene co-expression networks with Cytoscape software [74].

### Molecular evolution of genes involved in diosgenin saponin biosynthesis

To elucidate the molecular evolution of the *OSC* (PF13243), *CYP90B*, *CYP72A*, *CYP94*, *S3GT*, *F26G*, *CYP450* (PF0067), and *UGT* (PF00201) genes in *D*. *zingiberensis*, we first downloaded the hmm files of these gene families from Pfam (http://pfam.xfam.org/). Then, we identified *OSC*, *CYP450*, and *UGT* genes from the proteins of 13 *Dioscorea* plants and 93 other selected species using HMMER [75] software with the default parameters, and checked all candidate protein sequences using InterPro (http://www.ebi.ac.uk/interpro/search/sequence), SMART (http://smart.embl-heidelberg.de/), and the NCBI Conserved Domain Database (https://www.ncbi.nlm.nih.gov/Structure/cdd). Second, we used the protein sequences of *CAS*, *S3GT*, and *F26G* downloaded from NCBI database to identify homologous genes of all selected plants by BLASTP (E-value ≤1e−10, identity ≥50%). The *CYP90B*, *CYP94*, and *CYP72A* gene families were defined by the CYP450 database [76].

Finally, phylogenetic trees for all homologous genes were constructed with RAxML [66]. To detect possible tandem duplications in each gene family within this pathway, we extracted and analyzed the locations of all pathway genes on the chromosomes of the *D*. *zingiberensis* genome. We used the PAML [67] program to calculate the *K*_s_ values of all duplicated genes and estimated the divergence time of each diosgenin saponin biosynthetic pathway gene in *Dioscorea* plants using the formula *T* = *K*_s_/2*r*, where *T* represents the divergence time and *r* represents the synonymous substitution rate; the synonymous substitution rate of *Dioscorea* is 4.19 × 10^−9^ mutations per site per year.

### Determination of diosgenin and diosgenin saponin contents of the 13 *Dioscorea* species

To determine diosgenin saponin and diosgenin contents in *Dioscorea* plants, the metabolites were extracted from the fresh and young leaves from all 13 *Dioscorea* species with reference to the established protocol [18, 24, 47]. The relative contents of diosgenin and diosgenin saponins in different *Dioscorea* species were measured by LC–MS as described below.

To determine diosgenin saponin levels in *Dioscorea* species, we homogenized frozen leaf tissues with a chilled mortar and pestle, and then suspended 100 mg of the homogenate in 1.5 ml of 80% ethanol containing 0.04 mg/ml ginsenoside Rb1 (internal standard). Samples were sonicated in ice water for 30 minutes, held for at least 4 hours at 4°C, and centrifuged at 13 000 g for 15 minutes to remove precipitates. Finally, the supernatant was transferred to glass sample vials. The diosgenin saponin content in extracts was determined with a Vanquish ultra-performance liquid chromatography (UPLC) system (Thermo Scientific), which was equipped with a Hypersil GOLD C18 column (2.1 mm × 100 mm, 3 μm, Thermo Scientific) and coupled to a Q Exactive Hybrid Quadrupole-Orbitrap Mass Spectrometer (Q Exactive MS/MS, Thermo Scientific).

We extracted diosgenin from tissues using an established protocol with some modifications [24]. Briefly, 1 ml isopropanol was added to 100 mg homogenate from leaf samples, and homogenized thoroughly. These samples were extracted at 50°C with shaking at 200 rpm for 30 minutes, followed by two rounds of sonication for 20 minutes for further extraction. The supernatant was centrifuged at 13 000 g for 10 minutes, and analyzed using UPLC–MS/MS (Thermo Scientific). All raw data were processed using XCalibur software (Thermo Scientific). The metabolite content values were normalized using the *z*-score method and visualized using the pheatmap package (https://cran.r-project.org/web/packages/pheatmap/index.html) of R software.

### Comparative transcriptomic analysis of *Dioscorea* species

High-quality RNA was extracted from fresh leaves of the 13 *Dioscorea* plants. Sequencing libraries of each sample were generated using the NEBNext^®^ Ultra™ RNA Library Prep Kit for Illumina (NEB) according to the manufacturer’s instructions. Then, libraries were sequenced using the Illumina Hiseq 2500 platform. After raw sequencing data were filtered, a total of ~252 Gb of clean data containing 125- to 150-bp paired-end reads were generated for transcriptome assembly and gene functional annotation.

All clean paired-end reads were first mapped to our assembled *D*. *zingiberensis* genome using STAR [72]. The mapping rates of other *Dioscorea* plants, which were <70%, were analyzed using the *de novo* transcriptome assembly strategy. Transcriptome assembly was accomplished using Trinity [77]. Clean reads for these *Dioscorea* species were mapped back to their own *de novo* assembled transcriptomes, and the expression of each transcript in each sample was quantified using the RSEM software package [78]. The FPKM value of each gene was calculated based on the read counts obtained from RSEM results.

To investigate the expression levels of diosgenin saponin biosynthetic pathway genes in all 13 selected *Dioscorea* species, we used BLASTP (E-value <1e−10) to identify the homologous genes involved in the biosynthesis of diosgenin saponins. Then, we selected the best-hit homologous pathway genes in each *Dioscorea* plant and visualized them using the R package pheatmap (https://cran.r-project.org/web/packages/pheatmap/index.html).

### Ancestral state reconstructions and phylogenetic signal analysis

For ancestral state reconstruction, the presence/absence of diosgenin saponins was treated as the binary chemical trait, and the content of diosgenin saponins was treated as a continuous trait. In addition, we also collected the phenotypic data of diosgenin saponin for a total of 91 *Dioscorea* species and converted them into binary data [18, 22], and constructed the phylogenetic relationships using RAxML with 1000 bootstrap replicates [66, 79]. We then reconstructed ancestral states for each data set using the R package APE [80], and visualized them using the R package phytools [81].

To estimate the phylogenetic signal, the function phylosig of the phytools package was used to estimate the value of *λ* and *K* for continuous traits and binary traits [82, 83]. Based on Blomberg’s *K* statistic, *K* > 1 indicates that the phylogenetic signal is strong, and the similarity between close relatives is higher than expected under a Brownian threshold model [83]. *λ* or *K* ≈ 0 indicates that the trait distribution is phylogenetically random, suggesting that there is no correlation in the direction of evolution. On the contrary, *λ* or *K* ≈ 1 means non-random trait distribution.

Further details of the methods are provided in Supplementary Information.

## Acknowledgements

We thank Professors Jianbo Wang, Huabin Zhao, Yu Zhou (College of Life Sciences, Wuhan University), Professor Hui Ye (Department of Biology, Loyola University Chicago), and Professor Kai Ye (School of Electronics and Information Engineering, Xi’an Jiaotong University) for critical reading and valuable suggestions on this manuscript; Professor Yingjun Zhang (State Key Laboratory of Phytochemistry and Plant Resources in West China, Kunming Institute of Botany, Chinese Academy of Sciences) for suggestions on the chemical structure of metabolites; and Novogene Corporation for sequencing and preliminary analysis of transcriptome data. This work was supported by grants from the National Natural Science Foundation of China (30370152, 31270345, and 31470388, all to J.L.); and D.R.G.’s involvement was supported in part by the USDA National Institute of Food and Agriculture, Hatch project 227700.

## Author contributions

J.L. conceived and supervised the project; J.L. and Y.L. designed the experiments; Y.L. performed most of the experiments; C.T., Z.L., J.G., S.L., X.C., C.W., X.D., H.Y., W.S., L.H., Z.T., A.X., X.Y., W.W., Z.S., K.W., B.P., Y.B., and F.L. performed some of the experiments; Y.L., Z.L., S.L., X.D., H.Y., L.H., J.X., Z.T., X.Y., Z.S., and K.W. analyzed data; Y.L., Q.Y., L.C., J.C., X.Q., D.R.G., J.W., and J.L. wrote the manuscript. All authors read and approved the manuscript.

## Data availability

All assembled sequence and annotation data of the *D. zingiberensis* genome have been deposited in the NCBI database as a BioProject under accession number PRJNA716093. The RNA-seq raw data described in this paper have been deposited in the National Genomics Data Center, Beijing Institute of Genomics (China National Center for Bioinformation), Chinese Academy of Sciences, under the BioProject accession number PRJCA009925 and the Genome Sequence Archive (GSA) under accession number CRA007170 (https://bigd.big.ac.cn/gsa).

## Conflict of interest

The authors declare that they have no conflict of interest.

## Supplementary data

Supplementary data is available at *Horticulture Research* online.

## Supplementary Material

Web_Material_uhac165
