# Supplementary material for: The genome of Dioscorea zingiberensis sheds light on the biosynthesis, origin and evolution of the medicinally important diosgenin saponins
Source: Hortic Res. 2022 Jul 25;9:uhac165. doi: 10.1093/hr/uhac165 (PMC9531337; doi:10.1093/hr/uhac165)
Supplement: Web_Material_uhac165 [file web_material_uhac165.zip › Supplementary_Information_revised.docx]

**Supplementary** **Information for**

**Title:** **The genome of *Dioscorea zingiberensis* sheds light on the biosynthesis, origin and evolution of the medicinally important diosgenin saponins**

**Running title: Biosynthesis and evolution of diosgenin saponins**

Yi Li^1^, Chao Tan^1^, Zihao Li^1^, Jingzhe Guo^1^, Song Li^1^, Xin Chen^1^, Chen Wang^1^, Xiaokang Dai^1^, Huan Yang^1^, Wei Song^1^, Lixiu Hou^1^, Jiali Xu^1^, Ziyu Tong^1^, Anran Xu^1^, Xincheng Yuan^1^, Weipeng Wang^1^, Qingyong Yang^2^, Lingling Chen^3^, Zongyi Sun^4^, Kai Wang^4^, Bo Pan^5^, Jianghua Chen^6^, Yinghua Bao^7^, Faguang Liu^7^, Xiaoquan Qi^8^, David R. Gang^9^, Jun Wen^10^, Jiaru Li^1^

Correspondence: Jiaru Li ([jrli@whu.edu.cn](mailto:jrli@whu.edu.cn))

This supplemental file includes:

**Supplementary Materials and Methods**

**Supplementary figures:** Supplementary Fig. 1 to 16

**Supplementary Materials and Methods**

**Section S1 Genome sequencing, assembly, and annotation**

**1.1 Plant materials**

The seeds of *Dioscorea zingiberensis* were collected from Shiyan City, Hubei Province, China, and cultivated in the greenhouse of Wuhan University in October 2015. To harvest enough plant tissue for genome sequencing, we collected leaves, stems, and rhizomes in July 2018. Fresh tender leaves, stems, and rhizomes of *D*. *zingiberensis* were harvested and immediately frozen in liquid nitrogen, followed by preservation at -80°C prior to genome sequencing, RNA-seq, and metabolites analysis.

**1.2 DNA extraction, library preparation, and sequencing**

Genomic DNA was extracted from tender leaves of *D*. *zingiberensis* with the QIAGEN Genomic DNA extraction kit (QIAGEN) according to the protocol provided by the manufacturer. Then, the extracted genomic DNA was detected and quantified using a NanoDrop One UV-Vis spectrophotometer (Thermo Scientific) and Qubit 3.0 Fluorometer (Invitrogen, USA), respectively. Once the sample quality was assessed, the long DNA fragments were recovered using the BluePippin system (Sage Science). After purification, the ends of the DNA fragments were repaired, and the connection reaction was performed using the SQK-LSK109 kit. Then, the constructed DNA library was accurately quantified using the Qubit 3.0 Fluorometer (Invitrogen). Finally, the DNA libraries were loaded into the flow cell, and sequenced using Nanopore GridION X5 sequencer (Oxford Nanopore Technologies). The genomic DNA was also prepared for the 10× Genomics libraries, and sequenced on the Illumina Hiseq XTen platform.

**1.3 RNA extraction and sequencing**

Total RNA was extracted from each sample using the RNA Prep Pure Plant Kit. Sequencing libraries for each sample were constructed using the NEBNext® UltraTM RNA Library Prep Kit for Illumina (NEB) according to the manufacturer’s instructions, and index codes were added to attribute sequences to each sample. Similarly, clustering of the index-coded samples was performed on a cBot Cluster Generation System using the TruSeq PE Cluster Kit v3-cBot-HS (Illumina) according to the manufacturer’s instructions. After cluster generation, the library was sequenced on the Illumina Hiseq platform and 125-bp to 150-bp paired-end reads were generated (**Supplementary Table 9**).

**1.4 Hi-C library construction and sequencing**

The Hi-C library was prepared using an optimised protocol ^1^. Briefly, freshly collected tender leaves were cut into pieces and vacuum infiltrated in nuclear isolation buffer containing 2% formaldehyde. Crosslinking was prevented through the addition of glycine and additional vacuum infiltration. The fixed tissue was frozen in liquid nitrogen, ground to a powder, and suspended in nuclear isolation buffer to obtain a suspension. The purified nuclei were digested with 100 units of HindIII and incubated with biotin-14-dCTP. Due to the exonuclease activity of T4 DNA polymerase, biotin-14-dCTP was removed from non-ligated DNA ends. The ligated DNA was sheared into 300−600 bp fragments, and then repaired to blunt ends and A-tailed, followed by purification through biotin-streptavidin-mediated pull down. Finally, the Hi-C libraries were quantified and sequenced using the Illumina Hiseq platform (Illumina) (**Supplementary Table 1**).

**1.5 Estimation of genome size**

To estimate the genome size and heterozygosity of *D*. *zingiberensis*, we performed *K*-mer analysis using KMC and GenomeScope^2,3^ with 350 bp of Illumina paired-end reads. First, we plotted the *K*-mer frequency distribution, which showed two main peaks (**Supplementary Fig. 2a**). Then, we calculated the genome size based on the formula: G = *K*-mer number/*K*-mer depth, where G is the genome size, the *K*-mer number is the total number of *K*-mers (*K* = 17), and *K*-mer depth is estimated from the *K*-mer distribution. Finally, we estimated that the *D*. *zingiberensis* genome size is approximately 716 Mb with a 1.56% heterozygous rate.

1.6 Genome assembly

All Oxford Nanopore sequencing data were corrected using Nextdenovo software (<https://github.com/Nextomics/NextDenovo>). And then, SMARTdenovo (https://github.com/ruanjue/smartdenovo) was used for genome assembly. To further improve the accuracy of genome assembly, three rounds of consensus correction were performed using Illumina reads mapped using minimap2^4^ and Nextpolish^5^. To obtain optimal assembly, 10× Genomics data was filtered using fastp^6^, and aligned to filled gaps by ARKS^7^. Then, we used LINKS for scaffolding^8^. Based on the results of previous genomic survey analysis, the *D. zingiberensis* genome was high heterozygosity, resulting a large assembled genome. We removed the redundant sequence of the genome based on the interaction signal of Hi-C, and acquired the assembly of 629.18 Mb *D. zingiberensis* with a scaffold N50 length of 55.78 Mb (**Supplementary Table 2**).

To evaluate the completeness of the genome assembly, we used the BUSCO gene set^9^ to evaluate the completeness and accuracy of the *D. zingiberensis* genome. Of 1,614 BUSCO groups, we identified 1,563 complete BUSCOs (96.84%) in the *D. zingiberensis* genome. We also used CEGMA^10^ to predict the genome, and a total of 243 (97.98%) core genes were identified (**Supplementary Table 5**), suggesting that the genome assembly is nearly complete.

**1.7 Assembly with LACHESIS**

In total, 1,378,845,320 raw paired-end reads were generated from the Hi-C library. Then, quality control of the raw Hi-C data was performed using Hi-C-Pro (v2.8.0)^11^, as described previously. First, we used fastp (v0.12.6)^12^ to filter the raw reads (shorter than 30 bp) generated from Hi-C libraries, obtaining a total of 1,351 million high-quality clean reads, and then the high-quality clean paired-end reads were mapped to the draft assembled sequence using bowtie (v2.3.2)^13^ to obtain unique mapped paired-end reads. Finally, 288 million unique mapped paired-end reads were generated, of which 58.27% represented valid interaction pairs. Subsequently, we combined the valid Hi-C data and used the *de novo* assembly pipeline in LACHESIS^11^ software to produce chromosome groups. Briefly, the chromosome groups were produced based on hierarchical clustering using LACHESIS^11^. According to the interaction intensity of each contig, we constructed trunks, and selected the most reliable trunks for correcting the contig order. Finally, accurate contig locations in the genome were obtained (**Supplementary Fig. 3**, **Supplementary Table 3**). To verify the accuracy of assembly, we analysed the density distribution of genes and transposable elements (TEs) on the chromosomes, and the results are shown in **Fig. 2a**.

**Section S2 Genome annotation**

**2.1 Annotation of repeat sequences**

For repeat sequence annotation, LTR_FINDER^14^, MITE-Hunter^15^, and RepeatModeler (www.repeatmasker.org/RepeatModeler/) were used to construct a repeat database of this plant based on specific repeat sequence structures, which was merged with the Repbase^16^ database to form a comprehensive repeat sequence database, and finally RepeatMasker software (http://www.repeatmasker.org/) was used with that database to predict repeat sequences in the *D*. *zingiberensis* genome. We obtained a total of 378,551,220 bp of repeat sequences, accounting for 60.17% of the genome (**Supplementary Table 6**).

**2.2 Gene structure prediction and function annotation**

Gene structure prediction was conducted based on *ab initio* gene prediction, homologous protein prediction, and transcriptome data from three tissues of *D*. *zingiberensis*. *De novo* gene structure prediction was performed using Augustus^17^ software. For homologous protein prediction, GeMoMa^18^ was used to identify homologous proteins using the genomes of *Arabidopsis thaliana* ([http://plants.ensembl.org/*Arabidopsis_thaliana*/Info/Annotation/](http://plants.ensembl.org/Arabidopsis_thaliana/Info/Annotation/)), *Apostasia shenzhenica*^19^, *Dendrobium officinale*^20^, *Oryza sativa* ([http://plants.ensembl.org/*Oryza_sativa*/Info/Annotation/](http://plants.ensembl.org/Oryza_sativa/Info/Annotation/)), *Phalaenopsis equestris*^21^, and *Zostera marina*^22^, and the predicted results were filtered based on BUSCO assessment and integration. Then, PASA^23^ and TransDecoder (http://transdecoder.github.io/) were used to predict unigene sequences using the transcriptome data. The results predicted using the three methods above were integrated, and a total of 30,322 protein-coding gene sequences were predicted in the *D*. *zingiberensis* genome (**Supplementary Table 7**). Finally, these protein-coding gene models were functionally annotated by performing BLASTP searches against the common protein databases KEGG^24^, KOG^25^, SwissProt^26^, NR^27^, and GO^28^, and InterProScan^29^ was used to predict the conserved sequence and structure domains, generating 30,322 genes (**Supplementary Table 8**).

**Section S3 Evolutionary analysis of gene families**

Using the OrthoMCL^30^ pipeline, we identified gene families from *D*. *zingiberensis* and twelve other plants, including *Arabidopsis thaliana*^31^ (TAIR 10.0), *Artemisia annua*^32^, *Asparagus officinalis*^33^, *Amborella trichopoda*^34^, *Brachypodium distachyon*^35^ (v3.0), *Dioscorea rotundata*^36^, *Oryza sativa*^37^ (IRGSP-1.0.42), *Phoenix dactylifera*^38^, *Populus trichocarpa*^39^ (v3.0), *Salvia miltiorrhiza*^40^, *Solanum lycopersicum*^41^, and *Vitis vinifera* ^42^. First, we downloaded genome and protein data from online genomic databases, filtered out alternative splicings, and retained the longest transcript for each gene. Then, all-versus-all alignment of these filtered protein sequences from the selected species was performed using BLASTP^43,44^ with E-value ≤ 1e^-5^ and all other parameters at their default settings. Finally, the Markov cluster algorithm (MCL)^45,46^ was used to identify homologous gene families in each species. In total, 24,832 genes in 13,144 gene families were identified in the *D*. *zingiberensis* genome, of which 745 gene families consisting of 2,251 genes were specific to *D*. *zingiberensis*; 430 single-copy gene families present in all ten selected plants were used to construct the phylogenetic tree (**Supplementary Tables 10, 11)**.

We used CAFÉ^47^ software with the default parameters to detect gene family expansion and contraction in these ten plants. This method simulates a random gene birth-and-death rate to predict the evolution of gene families in various phylogenetic branches. Through prediction of gene families and comparative analysis with ancestral data, we could determine whether gene families in various plant species have gained or lost genes. Using this method, we predicted a total of 4,051 expanded gene families and 867 contracted gene families in *D*. *zingiberensis* (**Supplementary Table 12**). We performed gene functional enrichment analysis of specific and expanded gene families in *D*. *zingiberensis* using KEGG and GO databases (**Supplementary Figs. 8, 10**).

**Section S4 Phylogenetic analysis**

According to the results of gene family analysis, we used 430 identified single-copy genes to reconstruct the phylogenetic relationships among *D*. *zingiberensis* and ten other plant species. First, the protein sequences of the single-copy genes were aligned using MAFFT^48^. Then, the coding sequences of the proteins were aligned and further filtered using Gblock^49^ based on the alignment of the proteins. Finally, the GTR+GAMMA model was used for phylogenetic analysis based on the RaxML^50^ method, and the bootstrap value was set to 1,000, using *A*. *trichopoda* as the outgroup species. Finally, MEGA was used to visualise the phylogenetic tree^51^. To estimate the divergence times of *D*. *zingiberensis* from the other ten plant species, 430 single-copy genes were input to the MCMCTREE program in PAML^52^.

**Section S5 Whole-genome duplication analysis**

The synonymous substitution rates (*Ks*) analysis and phylogenomic methods can be used to directly infer the age of WGD events, with *Ks* plots reflecting the *Ks* distributions of paralogous genes and each peak on such plots representing a putative whole-genome duplication event^53,54^. For detection of whole-genome duplication events, we used the FASTKs pipeline (https://github.com/mrmckain/FASTKs) to estimate the *Ks* of the paralogous gene pairs in the *D*. *zingiberensis*, *D*. *rotundata*, and *Asparagus officinalis* genomes. The protein sequences of these three plants were aligned using BLASTN with E-value < 1e^-40^. Then, the BLAST hits (more than 100 pairs) were used for further analysis. For each pair, protein sequences were aligned using MUSCLE^55^ and converted to codon-based alignments using PAL2NAL^56^. *Ks*, and the nonsynonymous substitution rate (*Ka*) were estimated using PAML^52^ with the paired sequence setting. *Ks* distribution plots were drawn using the R package phytools^57^ and mclust (<https://cran.rstudio.com/web/packages/mclust/index.html>) (**Supplementary Fig. 9**). The WGD analysis of 12 other *Dioscorea* species was performed by the same method (**Supplementary Fig. 16**).

**Section S6 Biosynthesis of diosgenin saponins in *D*. *zingiberensis***

**S6.1 Immunohistochemical localisation of diosgenin**

To investigate the spatial and temporal changes of diosgenin content in *D*. *zingiberensis*, we used our previously developed rabbit polyclonal antibody specific for diosgenin to determine the synthetic sites of diosgenin in leaves, stems and rhizomes by immunochemical tissue localization^58^. The specific experimental steps are as follows: Fresh tissues of *D*. *zingiberensis* were sectioned freehand and immediately fixed in PLP fixative (2% paraformaldehyde, 0.075 M lysine, 0.037 M sodium phosphate, and 0.01 M sodium periodate). Sections were fixed overnight at 4°C after a short vacuum. After rinsing in phosphate-buffered saline (PBS), the sections were blocked with 2% (w/v) casein for 60 min at room temperature. Anti-diosgenin rabbit polyclonal antibody was incubated with the sections for 5 h at room temperature. After washing five times with PBS, the sections were incubated with alkaline phosphatase-conjugated goat anti-rabbit IgG (diluted 1:500 in blocking solution) for 3 h at room temperature. After washing with PBS, the sections were briefly rinsed with detection buffer (0.1 M sodium chloride and 0.1 M Tris-HCl, pH 9.5), and signals were developed using NBT/BCIP substrates for 30 min. Finally, the sections were post-fixed in ethanol-acetic acid solution (3:1, v/v) for 8 h at room temperature, dehydrated using graded ethanol (70–100%), and transferred to xylene. The sections were mounted on glass slides using neutral balsam. Control slides, created by omitting primary or secondary antibody incubation, are shown in **Supplementary Fig. 11**.

**S6.2 Diosgenin transport assay**

Each mature *D*. *zingiberensis* stem was cut at a point 30 cm above the ground with a sharp knife, and each end was inserted into a flask containing 50 mL deionised water. For some samples of aboveground tissues, all leaves were removed to determine the contribution of new diosgenin synthesis in leaves. To inhibit active transport within the phloem, 2,4-dinitrophenol (DNP) was added to 50 mL deionised water in the flask, reaching a final concentration of 1.5 mM. After 3 days under a 12 h light/dark photoperiod, the solution in each flask was collected and concentrated to 3 mL through vacuum evaporation. Finally, we measured the amount of diosgenin in the solution.

**S6.3 Expression analysis of candidate genes related to diosgenin saponin biosynthesis**

Using our assembled reference genome of *D*. *zingiberensis*, the expression levels of genes involved in diosgenin saponin biosynthesis were analysed from the transcriptomes of three *D*. *zingiberensis* tissues. An index of the reference genome was constructed and clean paired-end reads were aligned to the reference genome using STAR^59^ (v2.5.1b). STAR employs the Maximal Mappable Prefix (MMP) method, which can generate precise mapping results for junction reads. Then, we used HTSeq^60^ v0.6.0 to obtain the read numbers mapped to each gene. The fragments per kilobase of exon per million fragments mapped (FPKM) of each gene was calculated based on the length of the gene and the reads count mapped to the gene. We identified diosgenin saponin biosynthesis pathway genes using the BLASTP^44^ program (version 2.2.26). In total, we identified 167 expressed genes involved in diosgenin saponins biosynthesis in three tissues of *D*. *zingiberensis* (**Supplementary Table 13**). The gene expression levels of three *D*. *zingiberensis* tissues were visualised using the “pheatmap” package (<https://cran.r-project.org/web/packages/pheatmap/index.html>) of R software. Our results suggest that the majority of diosgenin biosynthesis pathway genes were highly expressed in leaves, followed by stems, and had the lowest expression in rhizomes.

**Section S7 Comparative phytochemical and transcriptomic analysis of *Dioscorea* plant species**

**7.1 Plant sample collection**

In total, we collected 13 *Dioscorea* plant species, including five species in sect. *Stenophora* (*Dioscorea zingiberensis*, *D. deltoidea*, *D. panthaica*, and *D. nipponica*), three species in sect. *Enantiophyllum* (*D. alata*, *D. fordii*, and *D. opposita*), two species in sect. *Lasiophyton* (*D. arachidna* and *D. pentaphylla*), one species in sect. *Opsophyton* (*D. bulbifera*), one species in sect. *Combilium* (*D.a esculenta*), one species in the New World I clade (*D. composita*), and one species in the Malagasy clade (*D. sansibarensis*), from southern China (**Supplementary Table 14**), and planted these plants in the greenhouse of Wuhan University in May 2019. Tender leaf samples of all these *Dioscorea* plants were collected, quickly frozen in liquid nitrogen, and stored at -80°C.

**7.2 RNA extraction, sequencing, assembly, and quantification**

High-quality RNA was extracted from fresh tender leaves of 13 *Dioscorea* plants using the method described in S1.3. Sequencing libraries of each sample were generated using the NEBNext® UltraTM RNA Library Prep Kit for Illumina (NEB) according to the manufacturer’s instructions. Then, sequencing libraries were obtained using the Illumina Hiseq 2500 platform. After raw sequencing data was filtered, the clean data containing 125-bp to 150-bp paired-end reads were generated for use in transcriptome assembly and gene functional annotation.

All clean paired-end reads were aligned to our assembled *D*. *zingiberensis* genome using the MMP method in STAR^59^. The mapping rates of other *Dioscorea* plants, which were less than 70%, were analysed using the *de novo* transcriptome assembly strategy. Clean reads for these *Dioscorea* species were mapped back to their own *de novo* assembled transcriptomes, and the expression of each transcript in each sample was quantified using the RSEM software package^61^. The FPKM of each gene was calculated based on the read counts obtained from RSEM.

**7.3 Comparative transcriptomic analysis of genes involved in diosgenin saponin biosynthesis among 13 *Dioscorea* plant species**

To investigate the expression levels of diosgenin saponin biosynthesis pathway genes in *D*. *zingiberensis* and other *Dioscorea* species, we performed the homologous genes involved in the biosynthesis of diosgenin saponins using all-versus-all BlastP method (E value < 1E-10) based on the transcriptome data of 13 *Dioscorea* plants, using the pathway genes from *D*. *zingiberensis* as query sequences. We visualised the expression levels using the R package pheatmap (<https://cran.r-project.org/web/packages/pheatmap/index.html>). Our results showed that the copy numbers and expression levels of genes involved in diosgenin saponin biosynthesis vary among the 13 *Dioscorea* plant species tested.

**7.4 Extraction of diosgenin saponins and diosgenin**

Diosgenin saponins were extracted from *Dioscorea* samples according to the methods previously reported^62,63^. The leaves were ground into powder with liquid nitrogen, then 100 mg of the powder was weighed and transferred to a centrifuge tube, and 1.5 mL of 20°C pre-cooled 80% ethanol (Thermo Scientific, HPLC grade) containing 0.04 mg/mL ginsenoside Rb1 (internal standard) was added. After the extraction homogenate was shaken, it was sonicated in ice water for 30 min, repeated 3 times, and then allowed to stand for 4 hours. After that, the extracted homogenate was centrifuged at 13,000 g for 15 min at 4°C, and the centrifugation was repeated twice. Finally, transfer the supernatant to a new glass vial for ultra-performance liquid chromatography (UPLC) using a Thermo Q Exactive tandem mass spectrometry (MS/MS) instrument. Six replicates were prepared for each sample.

We extracted diosgenin from tissues using an established protocol with some modifications^64^. Specifically, plant leaves were frozen with liquid nitrogen and ground to a powder. Approximately 100 mg of the leaves was weighed, soothed with 1 mL isopropanol (Thermo Scientific, HPLC grade), and mixed well. Put the mixed homogenate at 50°C and shake at 200 rpm/min for 30 min. Subsequently, extraction with ultrasound for 20 min, and repeated twice. The samples were placed in a centrifuge and centrifuged at 13,000 g for 10 min, repeated twice. After centrifugation, transfer the supernatant from the centrifuge tube to a glass vial, and analysed using UPLC-MS/MS.

**7.5 Metabolic profiling**

For detection of diosgenin saponins, an aliquot of 5 μL of each sample was injected for qualitative analysis using the Vanquish UPLC system (Thermo Scientific), which was equipped with a Hypersil GOLD C18 column, (2.1 mm × 100 mm, 3 μm, Thermo Scientific) and coupled to a Q Exactive Hybrid Quadrupole-Orbitrap Mass Spectrometer (Q Exactive MS/MS, Thermo Scientific). The mobile phases were eluent A, water with 0.1% (v/v) formic acid, and eluent B, acetonitrile with 0.1% (v/v) formic acid. The gradient program was as follows: 0 to 2 min, isocratic 12% B; 2 to 8 min, linear gradient of 12% to 45% B; 8 to 10 min, isocratic 45% B; 10 to 15 min, linear gradient of 45% to 95% B; 15 to 19 min, isocratic 95% B; 19.1 min, 12% B; 19.1 to 21 min, isocratic 12% B, at a column temperature of 40°C and a flow rate of 0.3 mL/min.

The MS/MS data were acquired with the Q Exactive MS/MS (Thermo Scientific) in positive mode. To obtain the fragmentation pattern, the mass spectrometer was operated in full-scan mode (scan range: 150–2000 m/z; resolution, 70,000), and target-SIM mode with the inclusion list: Dioscin: m/z 869.5022, 891.4834; Gracillin: m/z 885.5037, 907.4858; Protodioscin: m/z 1031.5434, 1071.5402; Pseudoprotogracillin: m/z 1047.5557, 1069.5381, 901.4967; Pseudoprotodioscin: m/z 1034.5491, 1053.5298; Zingberensis newsaponins: m/z 1047.5366, 1069.5181, 883.4716; Protodeltonin: m/z 1047.5348, 1087.5256; Protogracillin: m/z 1047.5412, 1087.5331; Parvifloside: m/z 1249.5791, 1209.5881. Protobioside: m/z 925.4766, 885.4891. Other ion source parameters: ion source: HESI-II, spray voltage (+) at 3000 V, capillary temperature at 325°C, probe heater temperature at 350°C, AGC target: 3e^6^, isolation window: 2.0 m/z, Maximum IT: 200 ms.

To determine the diosgenin content, the LC–MS system described above was used, but with different analysis methods. The mobile phases were eluent A, water with 5 mM ammonium acetate, and eluent B, methanol with 5 mM ammonium acetate. The gradient program was as follows: 0 to 2 min, isocratic 65% B; 2 to 9 min, linear gradient of 65% to 99% B; 9 to 9.1 min, 99% to 65%; 9.1 min to 11 min, isocratic 65% B. The column temperature was set at 40 °C, and a flow rate was 0.3 mL/min.

For MS/MS analysis, the Q Exactive MS (Thermo Scientific) was operated in positive target-SIM mode with the following parameters: probe heater temp, 350°C; ion source, HESI-II; diosgenin precursor ion selection at 415.3 m/z in positive ion mode, fragmentation at 20 V, and product ion selection at 271.2 m/z. The programs XCalibur (Thermo Scientific) was used to process the raw data. The contents of diosgenin and diosgenin saponins were calculated by comparing the peak area of each metabolite relative to that of the internal standard. Finally, the content data was processed by z-score normalization, and visualized using the “pheatmap” package (<https://cran.r-project.org/web/packages/pheatmap/index.html>) in R software.

**Section S8 Molecular evolution of genes involved in diosgenin saponin biosynthesis pathways**

**8.1 Identification of candidate genes related to diosgenin saponin biosynthesis**

To investigate candidate genes involved in diosgenin saponin biosynthesis, we used a comprehensive approach to identify such genes in all plants. According to previous research, the key genes and gene families, such as *CAS*, *OSC*, *CYP72A*, *CYP90B*, *CYP94*, *S3GT*, *F26G*, *CYP450* and *UGT*, are involved in diosgenin saponin biosynthesis (**Supplementary Table 17**). First, sequences of these genes were downloaded from an *Arabidopsis* gene database (TAIR, <https://www.arabidopsis.org/>) for use as query sequences. Then, we obtained protein sequences from the proteomes of 94 plant species from the corresponding genome database websites, respectively. Next, the query protein sequences were individually searched for in *D*. *zingiberensis* using BLASTP (E-value ≤ 1e-10, identity ≥ 50%) and HMMER^65^ software (version 3.2.1) with the default parameters. Finally, we combined the results from the two programs, and checked all candidate protein sequences using InterPro (<http://www.ebi.ac.uk/interpro/search/sequence>), SMART ([http://smart.embl-heidelberg.de/smart/](http://smart.embl-heidelberg.de/smart/set_mode.cgi?NORMAL=1)), and the NCBI Conserved Domain Database (<https://www.ncbi.nlm.nih.gov/Structure/cdd>).

8.2 Evolution analyses of genes involved in the diosgenin saponin biosynthesis

After identifying homologous genes involved in diosgenin saponin biosynthesis in all analysed plants, we explored the evolution of the diosgenin saponin biosynthesis pathway in *D*. *zingiberensis* using a phylogeny-based approach. The protein sequences of all identified genes were aligned using MAFFT^48^ with the default parameters. Phylogenetic trees of the key genes in all analysed plant species were constructed using the RaxML^50^. Through phylogenetic analysis, we found that 14 gene families were scattered among branches of the phylogenetic trees, indicating that these gene families might have undergone duplication in the *D*. *zingiberensis* genome.

To further elucidate the evolution of the diosgenin saponin biosynthesis pathway in *D*. *zingiberensis*, we evaluated the gene duplication event and the divergence time of each gene in this pathway. To detect possible tandem duplications among gene families, we extracted and analysed the locations of all pathway genes on the chromosomes of the *D*. *zingiberensis* genome (**Supplementary Fig. 14, Supplementary Table 18**). Based on the divergence time between *D*. *zingiberensis* and *D*. *rotundata* and the *Ks* value of their orthologous gene pairs, the synonymous substitution rate of *Dioscorea* is 4.19 × 10^-9^ mutations per site per year (**Fig.** **2b**). We used the PAML^52^ program to calculate the *Ks* values of these duplicated genes, and then estimated the divergence time using the formula T = *Ks*/2r, where T represents the divergence time and r represents the synonymous substitution rate^66-68^. In the *D. zingiberensis* genome only, most of the pathway genes, especially the eleven key genes, were duplicated during the WGD events occurred approximately ~ 18 Ma and ~ 95 Ma **(Supplementary Fig. 9,** **Supplementary Table 19**), which was unique to *D. zingiberensis.*

In addition, we used Pearson’s correlation (value cut off |0.8|, *P* < 0.05) analyses to investigate whether significant correlations exist between the expression levels of genes in the diosgenin saponin biosynthesis pathway and contents of diosgenin saponins in *Dioscorea* plant species (**Fig. 6c**).

8.3 Ancestral states reconstructions and phylogenetic signal analysis

For the 91 *Dioscorea* species for which we collected diosgenin saponin data, we constructed the phylogenetic relationships of these species using the sequences of seven conserved genes, such as (18S rDNA, 26S rDNA, ITS, matK, rbcL, atpB, and trnL-F)^69^. RaXML was used to construct the maximum-likelihood (ML) phylogenetic tree with 1000 bootstrap replicates^50^, using *Asparagus officinalis* as the outgroup.

For ancestral states reconstruction, the presence/absence of diosgenin saponins was used to treated as the binary chemical trait, and the copy number of genes in the five pathways involved in the biosynthesis of diosgenin saponins were treat as continuous traits. And then, we fit and reconstructed ancestral states for each data set using the R package phytools^57^.

To estimate the phylogenetic signal, the function “phylosig” of phytools package was used to estimate the value of λ and *K* for continuous traits and binary traits^70,71^. Based on Blomberg`s K statistic, K > 1 indicates that the phylogenetic signal is strong, and the similarity between close relatives is higher than expected under a Brownian threshold model^71^. λ or K ≈ 0 indicates that the trait distribution is phylogenetically random, suggesting that there is no correlation in the direction of evolution. On the contrary, λ or K ≈ 1signify the non-random trait distribution (**Supplementary Tables 15, 16**).

**References**

1. Belton, J. M. *et al.* Hi-C: A comprehensive technique to capture the conformation of genomes. *Methods* **58**, 268–276 (2012).

2. Kokot, M., Dlugosz, M. & Deorowicz, S. KMC 3: counting and manipulating k-mer statistics. *Bioinformatics* **33**, 2759–2761 (2017).

3. Vurture, G. W., *et al.* GenomeScope: Fast reference-free genome profiling from short reads. *Bioinformatics* **33**, 2202–2204 (2017).

4. Li, H. Minimap and miniasm: Fast mapping and de novo assembly for noisy long sequences. *Bioinformatics* **32**, 2103–2110 (2016).

5. Hu, J., Fan, J., Sun, Z. & S. Liu, NextPolish: A fast and efficient genome polishing tool for long-read assembly. *Bioinformatics* **36**, 2253–2255 (2020).

6. Li, H. & Durbin, R. Fast and accurate short read alignment with Burrows-Wheeler transform. *Bioinformatics* **25**, 1754–1760 (2009).

7. Coombe, L. *et al.* ARKS: Chromosome-scale scaffolding of human genome drafts with linked read kmers. *bioRxiv*, 1–10 (2018).

8. Warren, R. L. *et al*. LINKS: Scalable, alignment-free scaffolding of draft genomes with long reads. *GigaScience* **4** (2015), doi:10.1186/s13742-015-0076-3.

9. Simão, F. A., Waterhouse, R. M., Ioannidis, P., Kriventseva, E. V. & Zdobnov, E. M. BUSCO: Assessing genome assembly and annotation completeness with single-copy orthologs. *Bioinformatics* **31**, 3210–3212 (2015).

10. Parra, G., Bradnam, K. & Korf, I. CEGMA: A pipeline to accurately annotate core genes in eukaryotic genomes. *Bioinformatics* **23**, 1061–1067 (2007).

11. Burton, J. N. *et al*. Chromosome-scale scaffolding of de novo genome assemblies based on chromatin interactions. *Nat. Biotechnol*. **31**, 1119–1125 (2013).

12. Chen, S., Zhou, Y., Chen, Y. & Gu, J. Fastp: An ultra-fast all-in-one FASTQ preprocessor. *Bioinformatics* **34**, i884–i890 (2018).

13. Langmead, B. & Salzberg, S. L. Fast gapped-read alignment with Bowtie 2. *Nat. Methods*. **9**, 357–359 (2012).

14. Xu, Z. & Wang, H. LTR-FINDER: An efficient tool for the prediction of full-length LTR retrotransposons. *Nucleic Acids Res*. **35**, 265–268 (2007).

15. Han, Y. & Wessler, S. R. MITE-Hunter: A program for discovering miniature inverted-repeat transposable elements from genomic sequences. *Nucleic Acids Res*. **38**, 1–8 (2010).

16. Bao, W., Kojima, K. K. & Kohany, O. Repbase Update, a database of repetitive elements in eukaryotic genomes. *Mobile DNA* **6**, 4–9 (2015).

17. Stanke, M., Schöffmann, O., Morgenstern, B. & Waack, S. Gene prediction in eukaryotes with a generalized hidden Markov model that uses hints from external sources. *BMC Bioinformatics* **7**, 1–11 (2006).

18. Keilwagen, J. *et al.* Using intron position conservation for homology-based gene prediction. *Nucleic Acids Res*. **44**, 1–11 (2016).

19. Zhang, G. Q. *et al.* The *Apostasia* genome and the evolution of orchids. *Nature* **549**, 379–383 (2017).

20. Yan, L. *et al.* The genome of *Dendrobium officinale* illuminates the biology of the important traditional Chinese orchid herb. *Mol. Plant* **8**, 922–934 (2015).

21. Cai, J. *et al.* The genome sequence of the orchid *Phalaenopsis equestris*. *Nat. genet*. **47**, 65–72 (2015).

22. Olsen, J. L. *et al*. The genome of the seagrass *Zostera marina* reveals angiosperm adaptation to the sea. *Nature* **530**, 331–335 (2016).

23. Haas, B. J. *et al*. Automated eukaryotic gene structure annotation using evidence modeler and the program to assemble spliced alignments. *Genome Biol*. **9**, 1–22 (2008).

24. Kanehisa, M. & Goto, S. KEGG: Kyoto Encyclopedia of Genes and Genomes. *Nucleic Acids Res*. **28**, 27–30 (2000).

25. Tatusov, R. L. *et al*. The COG database: An updated vesion includes eukaryotes. *BMC Bioinformatics* **4**, 1–14 (2003).

26. Bairoch, A. *et al*. The Universal Protein Resource (UniProt). *Nucleic Acids Res*. **33**, 154–159 (2005).

27. Pruitt, K. D. Tatusova, T. & Maglott, D. R. NCBI Reference Sequence (RefSeq): A curated non-redundant sequence database of genomes, transcripts and proteins. *Nucleic Acids Res*. **33**, 501–504 (2005).

28. Harris, M. A. *et al*. The Gene Oncology (GO) database and informatics resource. *Nucleic Acids Res*. **32**, 258–261 (2004).

29. Hunter, S. *et al.* InterPro: The integrative protein signature database. *Nucleic Acids Res*. **37**, 211–215 (2009).

30. Li, L., Stoeckert, C. J. J. & Roos, D. S. OrthoMCL: Identification of Ortholog Groups for Eukaryotic Genomes, *Genome Res*. **13**, 2178–2189 (2003).

31. The Arabidopsis Genome Initiative. Analysis of the genome sequence of the flowering plant *Arabidopsis thaliana*. *Nature* **408**, 796–815 (2000).

32. Shen, Q. *et al*. The genome of *Artemisia annua* provides insight into the evolution of asteraceae family and artemisinin biosynthesis. *Mol. Plant*. **11**, 776–788 (2018).

33. Harkess, A. *et al*. The asparagus genome sheds light on the origin and evolution of a young y chromosome. *Nat. Commun*. **8** (2017), doi:10.1038/s41467-017-01064-8.

34. DePamphilis, C. W. *et al*. The *Amborella* genome and the evolution of flowering plants. *Science* **342** (2013), doi:10.1126/science.1241089.

35. Vogel, J. P. et al. Genome sequencing and analysis of the model grass *Brachypodium distachyon*. *Nature* **463**, 763–768 (2010).

36. Tamiru, M. *et al*. Genome sequencing of the staple food crop white Guinea yam enables the development of a molecular marker for sex determination. *BMC Biology* **15**, 1–20 (2017).

37. Kawahara, Y. *et al*. Improvement of the *Oryza sativa Nipponbare* reference genome using next generation sequence and optical map data. *Rice* **6**, 1–10 (2013).

38. Hazzouri, K. M. *et al*. Genome-wide association mapping of date palm fruit traits. *Nat. Commun*. **10**, 1–14 (2019).

39. Tuskan, G. A. *et al*. The genome of black cottonwood, *Populus trichocarpa* (Torr. & Gray). *Science* **313**, 1596–1604 (2006).

40. Xu, H. *et al*. Analysis of the genome sequence of the medicinal plant *Salvia miltiorrhiza*. *Mol. Plant*. **9**, 949–952 (2016).

41. Sato, S. *et al*. The tomato genome sequence provides insights into fleshy fruit evolution. *Nature* **485**, 635–641 (2012).

42. Jaillon, O. *et al*. The grapevine genome sequence suggests ancestral hexaploidization in major angiosperm phyla. *Nature* **449**, 463–467 (2007).

43. Camacho, C. *et al*. BLAST+: Architecture and applications. *BMC Bioinformatics* **10**, 1–9 (2009).

44. Zeng, J. *et al*. Gapped BLAST and PSI-BLAST: a new generation of protein database search programs. *World J Microbiol Biotechnol*. **25**, 3389–3402 (2007).

45. Van Dongen, S. & Abreu-Goodger C., Using MCL to extract clusters from networks. *Methods Mol. Biol.* **804**, 281–295 (2012).

46. Enright, A. J., Van Dongen, S. & Ouzounis, C. A. An efficient algorithm for large-scale detection of protein families. *Nucleic Acids Res*. **30**, 1575–1584 (2002).

47. De Bie, T., Cristianini, N., Demuth, J. P. & Hahn, M. W. CAFE: A computational tool for the study of gene family evolution. *Bioinformatics* **22**, 1269–1271 (2006).

48. Katoh, K. & Standley, D. M. MAFFT multiple sequence alignment software version 7: Improvements in performance and usability. *Mol. Biol. Evol*. **30**, 772–780 (2013).

49. Castresana, J. Selection of conserved blocks from multiple alignments for their use in phylogenetic analysis. *Mol. Biol. Evol*. **17**, 540–552 (2000).

50. Stamatakis, A. RAxML version 8: A tool for phylogenetic analysis and post-analysis of large phylogenies. *Bioinformatic*s **30**, 1312–1313 (2014).

51. Tamura, K. Stecher, G. Peterson, D. Filipski, A. & Kumar, S. MEGA 6: Molecular Evolutionary Genetics Analysis Version 6.0. **30**, 2725–2729 (2013).

52. Yang, Z. PAML 4: Phylogenetic analysis by maximum likelihood. *Mol. Biol. Evol*. **24**, 1586–1591 (2007).

53. Unruh, S. A. *et al*. Phylotranscriptomic analysis and genome evolution of the Cypripedioideae (Orchidaceae). *Am. J. Bot*. **105**, 631–640 (2018).

54. Clark, J. W. & Donoghue, P. C. J. Whole-Genome Duplication and Plant Macroevolution. *Trends Plant Sci*. **23**, 933–945 (2018).

55. Edgar, R. C. MUSCLE: A multiple sequence alignment method with reduced time and space complexity. *BMC Bioinformatics* **5**, 1–19 (2004).

56. Suyama, M. Torrents, D. & Bork, P. PAL2NAL: Robust conversion of protein sequence alignments into the corresponding codon alignments. *Nucleic Acids Res*. **34**, 609–612 (2006).

57. Revell, L. J. phytools: An R package for phylogenetic comparative biology (and other things). *Methods Ecol. Evol*. **3**, 217–223 (2012).

58. Li, J., Yang, D., Yu, He, K. J. & Zhang, Y. Determination of diosgenin content in medicinal plants with enzyme-linked immunosorbent assay. *Planta Med*. **76**, 1915–1920 (2010).

59. Dobin, A. *et al*. STAR: Ultrafast universal RNA-seq aligner. *Bioinformatics.* **29**, 15–21 (2013).

60. Anders, S., Pyl, P. T. & Huber, W. HTSeq-A Python framework to work with high-throughput sequencing data. *Bioinformatics* **31**, 166–169 (2015).

61. Li, B. & Dewey, C. N. RSEM: Accurate transcript quantification from RNA-Seq data with or without a reference genome. *BMC Bioinformatics.* **12**, (2011), doi: 10.1186/1471-2105-12-323.

62. Li, X. *et al*. Chemotaxonomic studies of 12 *Dioscorea* species from China by UHPLC-QTOF-MS/MS analysis. *Phytochem. Anal*. **31**, 164–182 (2020).

63. Hou, L. *et al.* Genome‐wide identification of *CYP72A* gene family and expression patterns related to jasmonic acid treatment and steroidal saponin accumulation in *Dioscorea zingiberensis*. *Int. J. Mol. Sci.* **22**, (2021).

64. Christ, B. *et al*. Repeated evolution of cytochrome P450-mediated spiroketal steroid biosynthesis in plants. *Nat. Commun*. **10**, 1–11 (2019).

65. Johnson, L. S., Eddy, S. R. & Portugaly, E. Hidden Markov model speed heuristic and iterative HMM search procedure. *BMC Bioinformatics* **11** (2010), doi:10.1186/1471-2105-11-431.

66. Chen, C. *et al*. TBtools - an integrative toolkit developed for interactive analyses of big biological data. *Mol. Plant*. 1–9 (2020).

67. Wei, C. *et al*. Draft genome sequence of *Camellia sinensis* var. *sinensis* provides insights into the evolution of the tea genome and tea quality. *Proc. Natl. Acad. Sci. USA*. **115**, E4151–E4158 (2018).

68. Hu, L. et al. The chromosome-scale reference genome of black pepper provides insight into piperine biosynthesis. *Nat. Commun*. **10** (2019), doi:10.1038/s41467-019-12607-6.

69. Zanne, A. E. *et al*. Three keys to the radiation of angiosperms into freezing environments. *Nature* **506**, 89–92 (2014).

70. Pagel, M. Inferring evolutionary processes from phylogenies. *Zool. Scr*. **26**, 331–348 (1997).

71. Blomberg, S. P. Garland, T. & Ives, A. R. Testing for phylogenetic signal in comparative data: Behavioral traits are more labile. *Evolution* **57**, 717–745 (2003).

**Supplementary figures:**


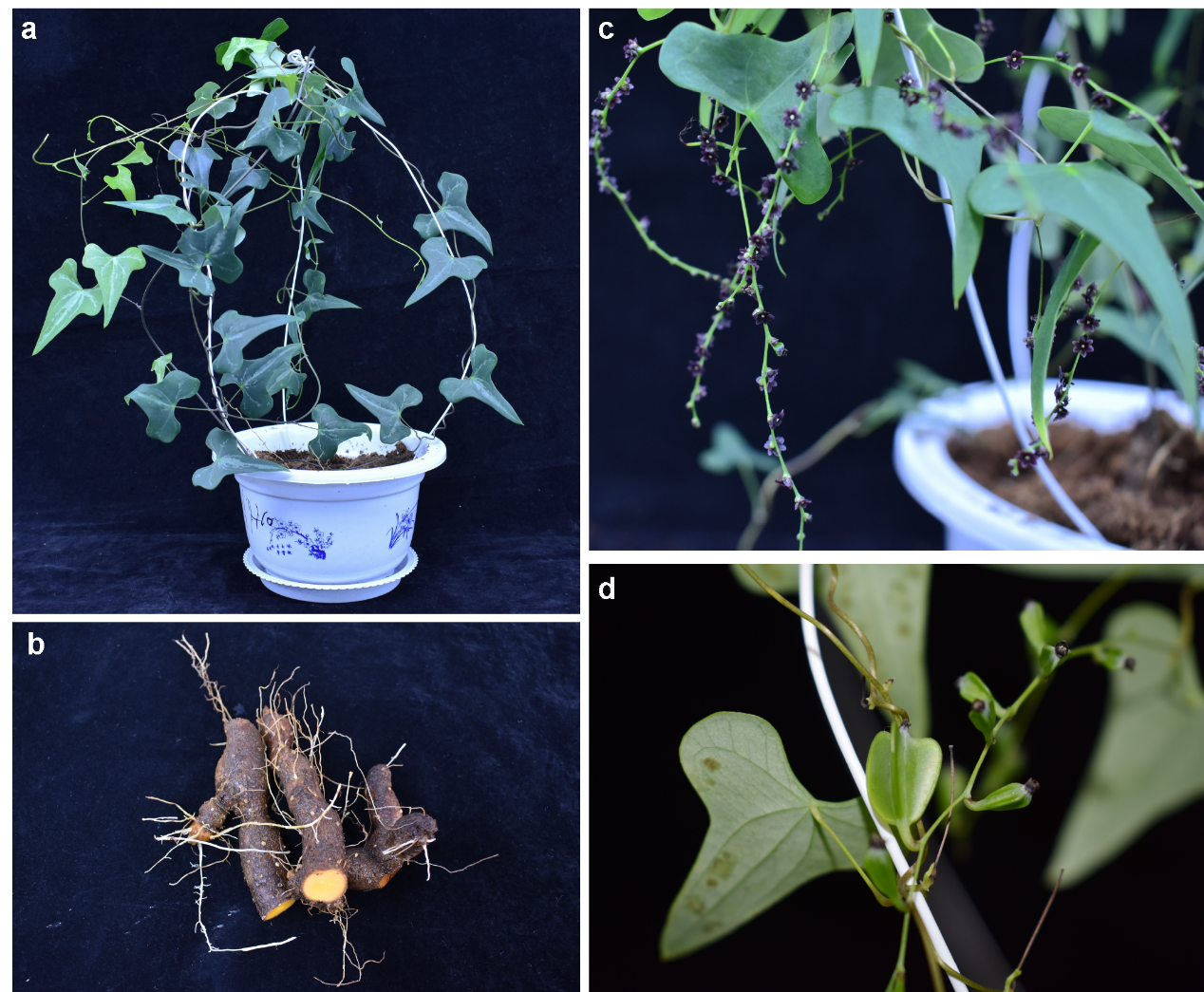


**Supplementary Fig. 1. Morphological characteristics of *D. zingiberensis*.** Pictures of the aerial parts (**a**), rhizomes (**b**), flowers (**c**), and capsules (**d**) of *D. zingiberensis* plants grown in the greenhouse of Wuhan University.


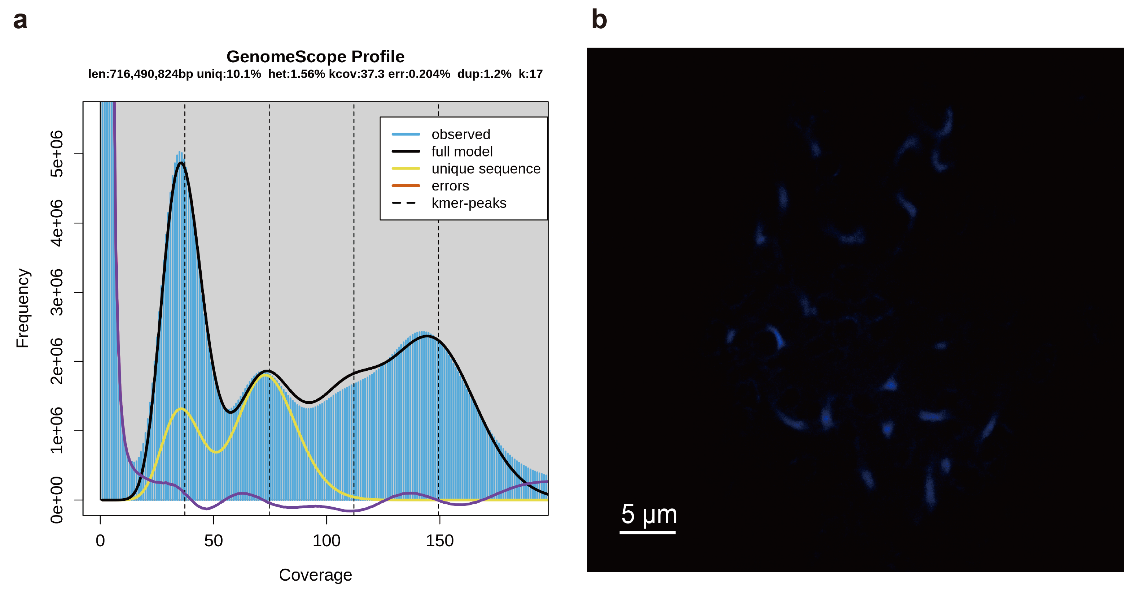


**Supplementary Fig. 2.** ***K*-mer frequency distribution and karyotype of *D. zingiberensis*.** (**a**) Graph of *K*-mer distribution; X axis shows *K*-mer depth and Y axis shows *K*-mer frequency. As *K*-mer depth follows a Poisson distribution, *K*-mer depth could be calculated from the graph, and the main *K*-mer peak volume was 37.3. The genome size was measured as 716 Mb. (**b**) *D. zingiberensis* shows 20 mitotic metaphase chromosomes in a root cell stained using fluorescent dye (DAPI).


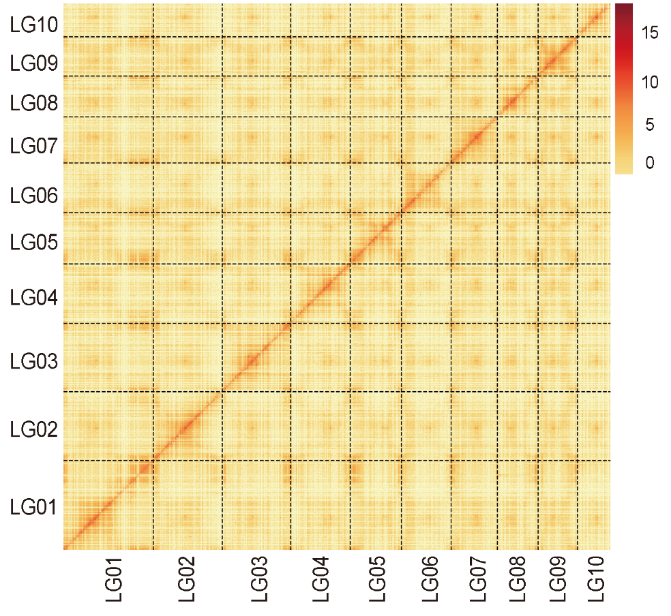


**Supplementary Fig. 3. Heatmap of Hi-C genome assembly.** Hi-C interactions among 10 chromosomes of the *D. zingiberensis* genome. LG01-LG10 indicates chromosome 1–10. X and Y axes indicate the order of scaffolds on the corresponding chromosomes.


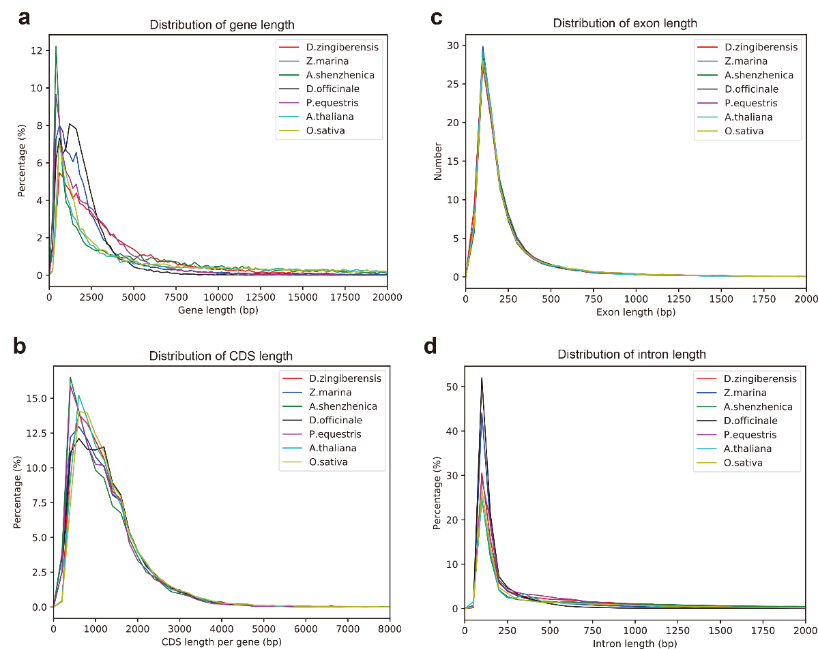


**Supplementary Fig. 4. Distribution map of gene element in plants.** The distribution of gene length (**a**), CDS length (**b**), exon length (**c**), and intron length (**d**) were shown in the plots, respectively. Each species corresponds to a color.


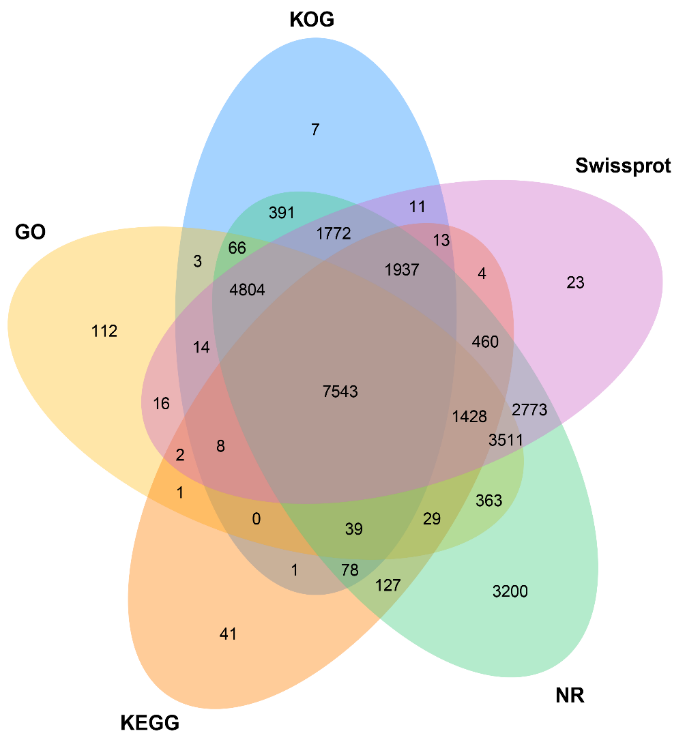


**Supplementary Fig. 5. Venn diagram of gene functional annotation databases.** The protein sequences obtained were compared with common functional databases including KEGG, KOG, SwissProt, and NR using BLASTP, and the corresponding annotation statistical results were as follows: KOG, 55.03%; KEGG, 38.62%; NR, 94.06%; SwissProt, 80.2%; and GO, 59.16%.


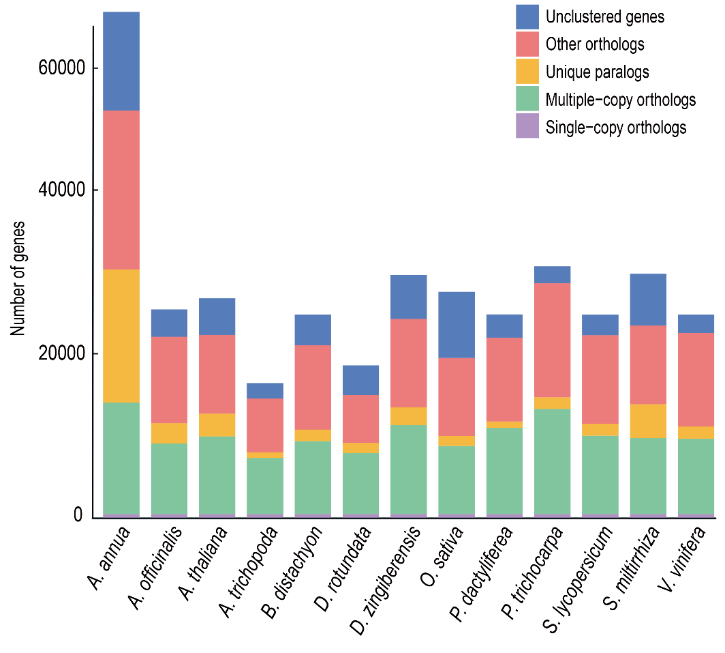


**Supplementary Fig. 6. Comparison of ortholog gene families among 13 plant species**. The y-axis shows the gene numbers of homologous gene families in the genome, and the x-axis shows the name of thirteen plant species, respectively.


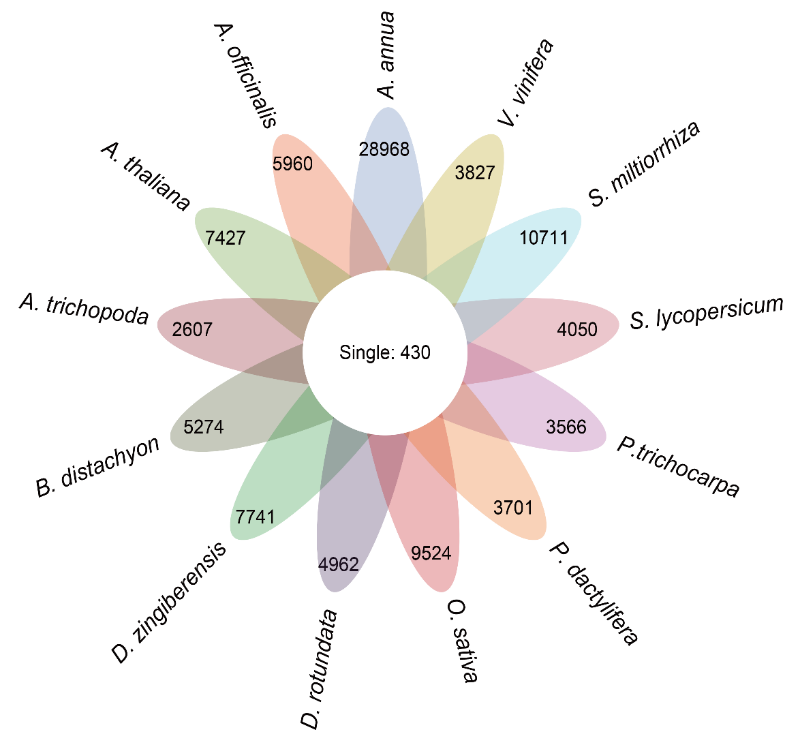


**Supplementary Fig. 7. Venn diagram of shared and unique genes among 13 plant species**. The corresponding number on the petal is the sum of unique and un-clustered genes in each species, and the number in the middle represents single-copy orthologous genes. The specific numbers are shown in **Supplementary** **Table 11**.


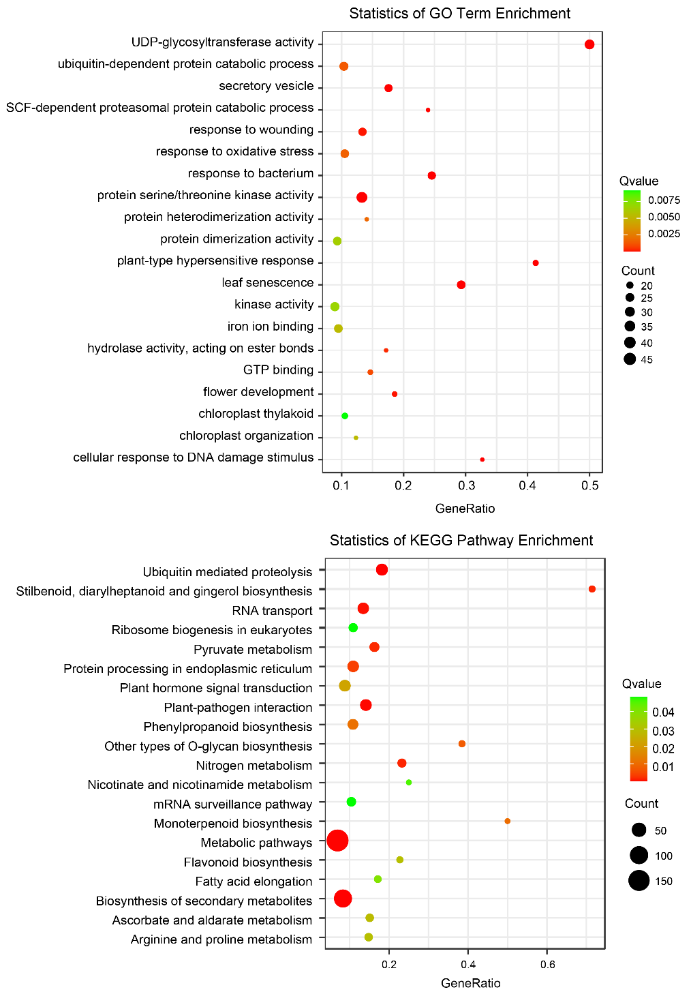


**Supplementary Fig. 8. KEGG and GO functional enrichment of *D*. *zingiberensis* unique gene families.** The two parts of the figure show KEGG and GO enrichment analysis results; the colors of bubbles in the figure correspond to the Q-value, and the size of bubbles represents the number of genes.


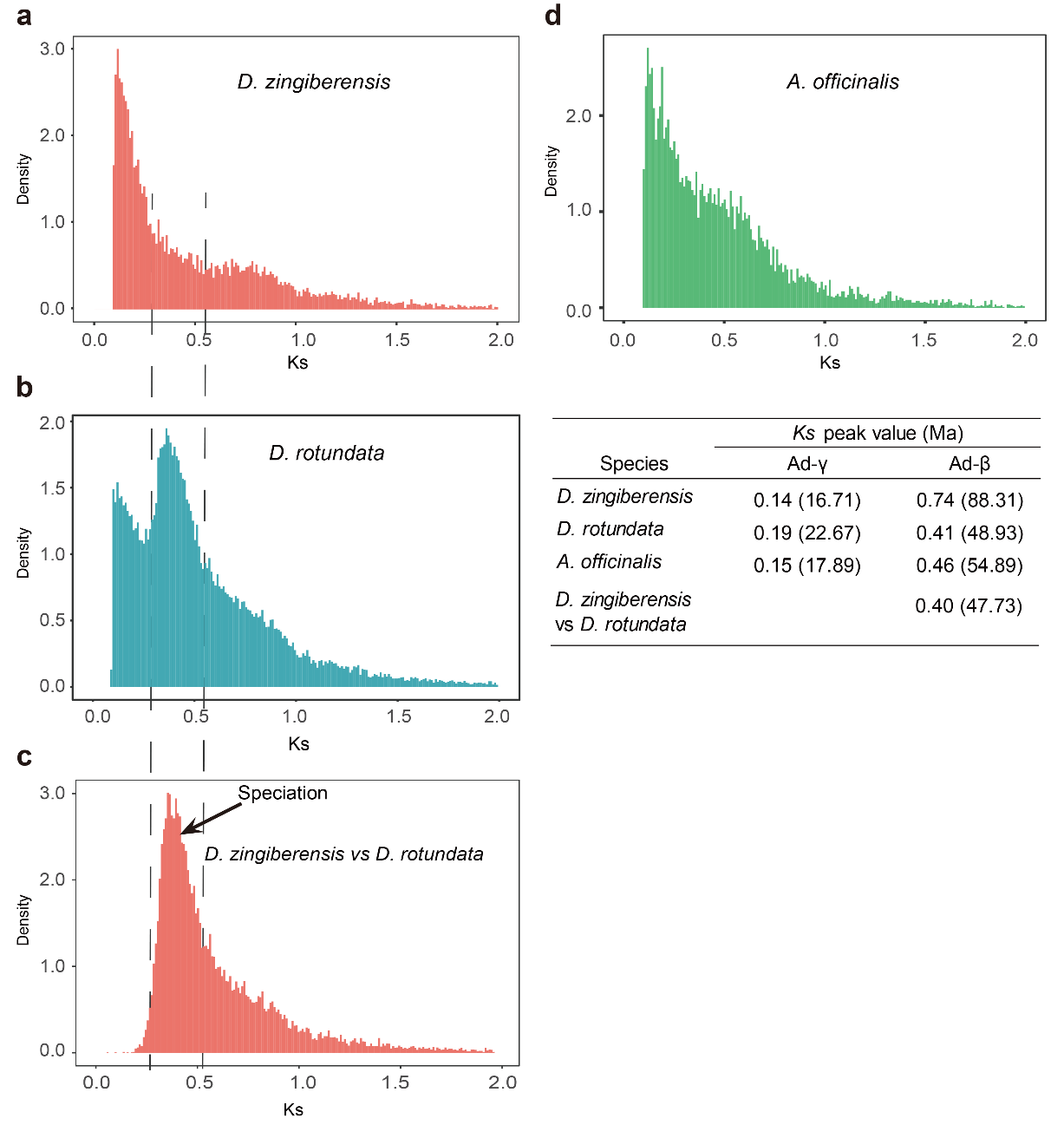


**Supplementary Fig. 9. The *Ks* distribution of paralogous gene pairs of 3 diosgenin saponin-containing species.** (**a**) *D. zingiberensis*, (**b**) *D. rotundata*, (**c**) speciation between *D. zingiberensis* and *D. rotundata*, (**d**) *A. officinalis*. Paralogous gene pairs for each species were generated by using the FASTKs pipeline. The *Ks* and the nonsynonymous substitution rate (*Ka*) were estimated using PAML. The *Ks* frequency plots were then drawn by R packages ggplot2 and Mclust.


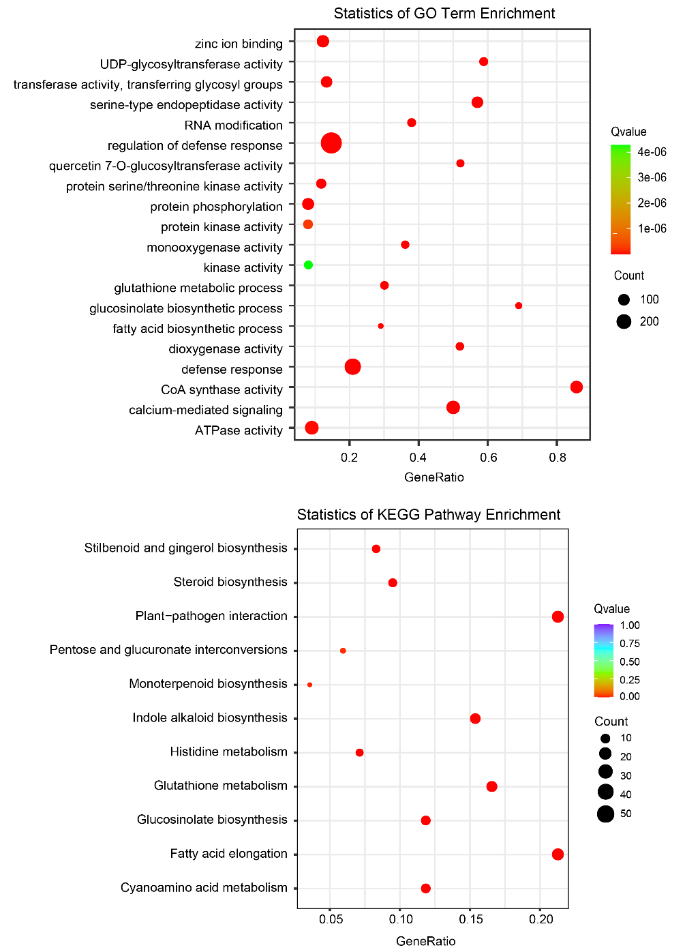


**Supplementary Fig. 10. KEGG and GO functional enrichment of *D*. *zingiberensis* expanded gene families.** The two parts of the figure show KEGG and GO enrichment analysis results; the colors of bubbles in the figure correspond to the Q-value, and the size of bubbles represents the number of genes.


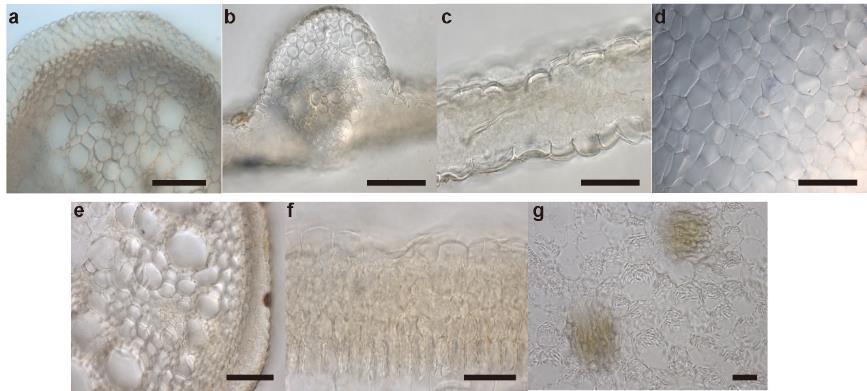


**Supplementary Fig. 11. Immunohistochemical localization of diosgenin in the stems, leaves, and rhizomes of *D*. *zingiberensis*.** Microsections without the addition of primary antibody (**a**–**d**) or secondary antibody (**e**–**g**) during immunolocalisation of diosgenin in the stem (**a** and **e**), leaf (**b**, **c**, and **f**), and rhizome (**d** and **g**) of *D*. *zingiberensis*. Bar =100 μm.


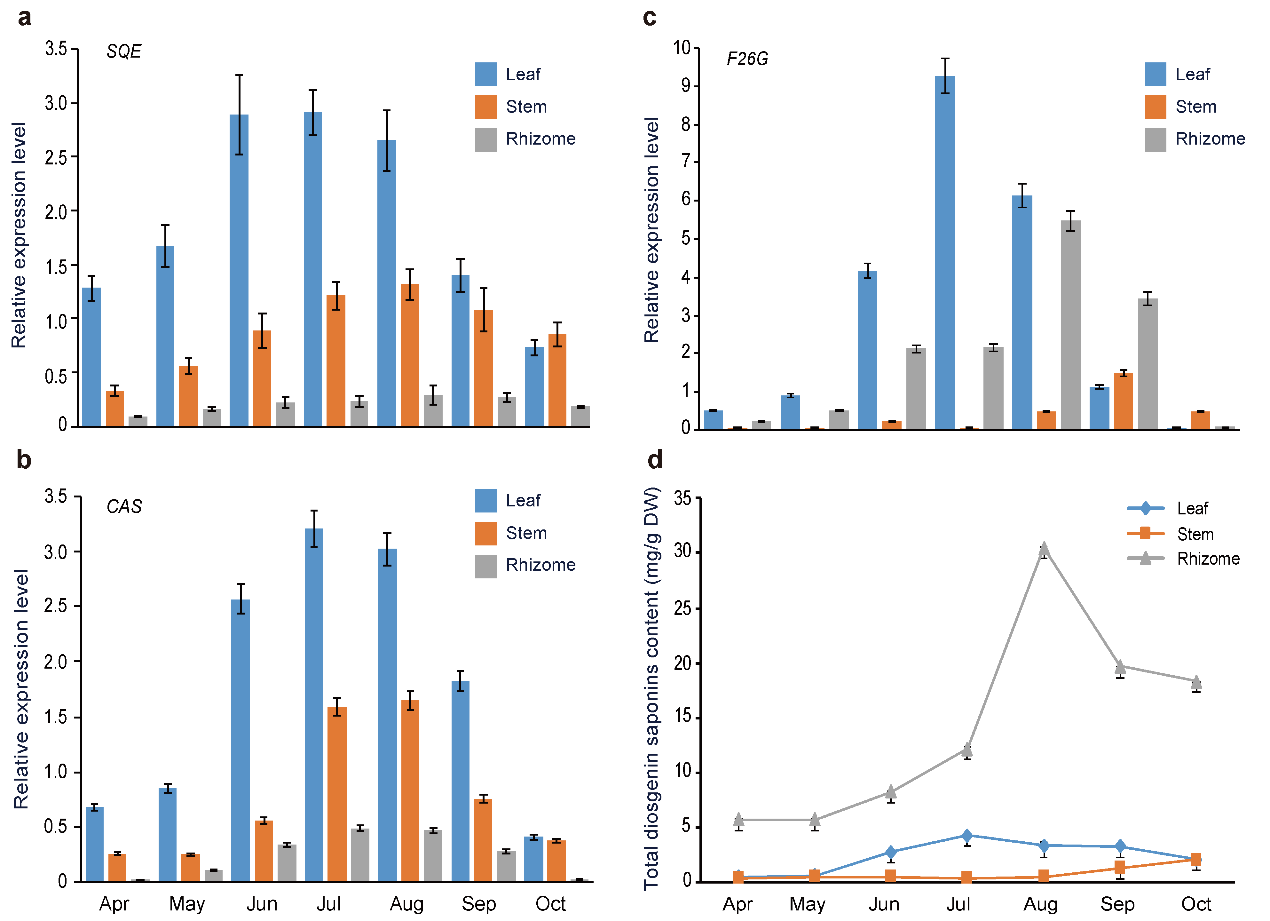


**Supplementary Fig. 12. Analysis of 3 key gene expression levels and total diosgenin saponins content in 3 tissues of *D*. *zingiberensis*.** Expression patterns of *SQE* (**a**), *CAS* (**b**), and *F26G* (**c**), and accumulation of total diosgenin saponins (**d**) in 3 tissues of *D*. *zingiberensis* during the development period. Relative expression levels of genes were measured using qRT–PCR. DW, dry weight. Three biological replicates for each sample were collected, and the error bars represented standard error.


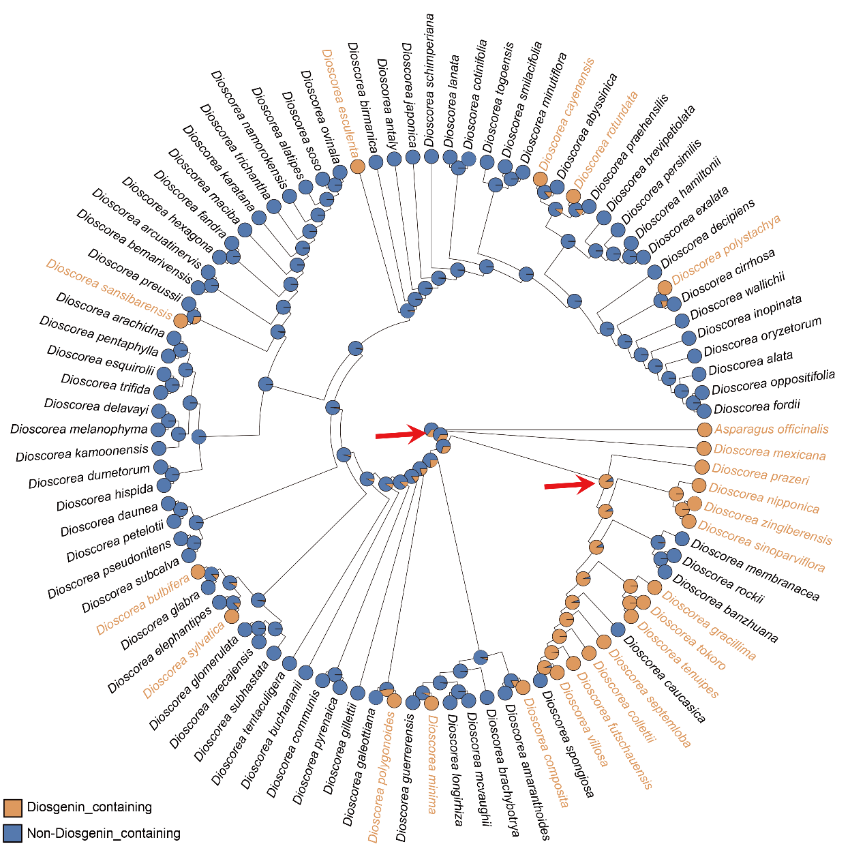


**Supplementary Fig. 13. Ancestral state reconstruction of diosgenin saponin phenotype in *Dioscorea* species.** Species name marked in brown represent species with diosgenin saponins, those marked in wathet represent species without diosgenin saponins. The red arrows indicate the evaluated ancestral nodes.


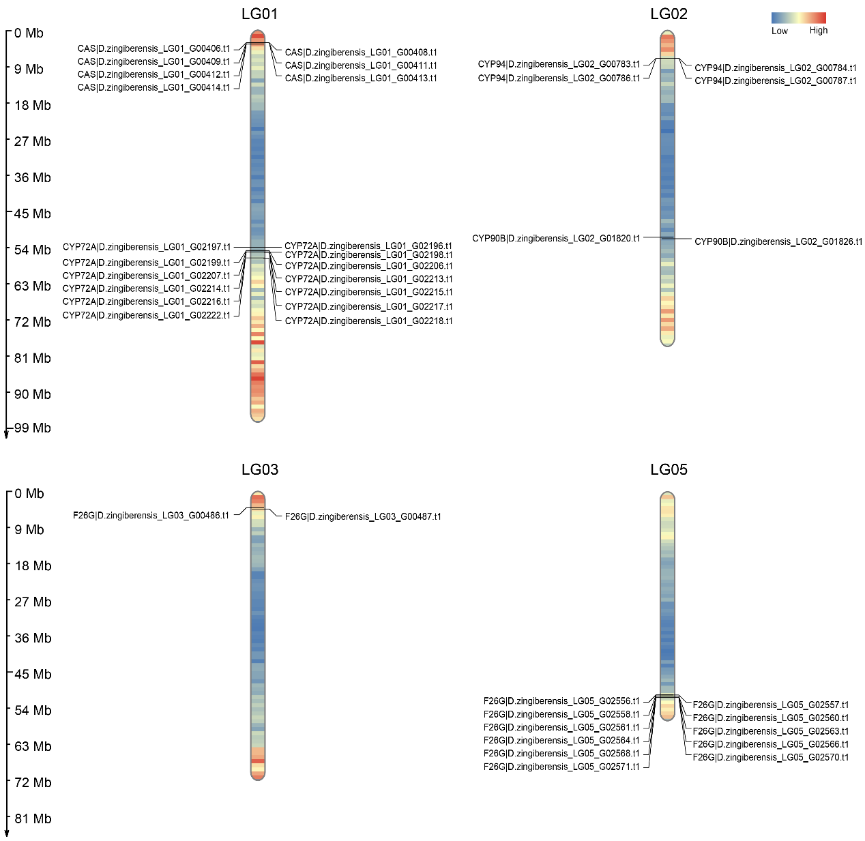


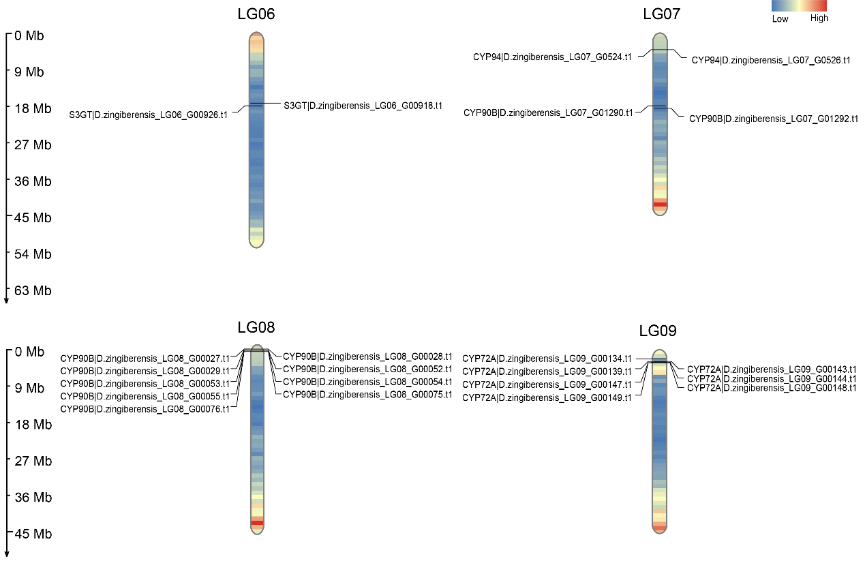


**Supplementary Fig. 14. Chromosome position of tandem duplication genes involved in diosgenin saponins biosynthesis in the *D. zingiberensis* genome.** The color of chromosomes indicates the density of genes.

**
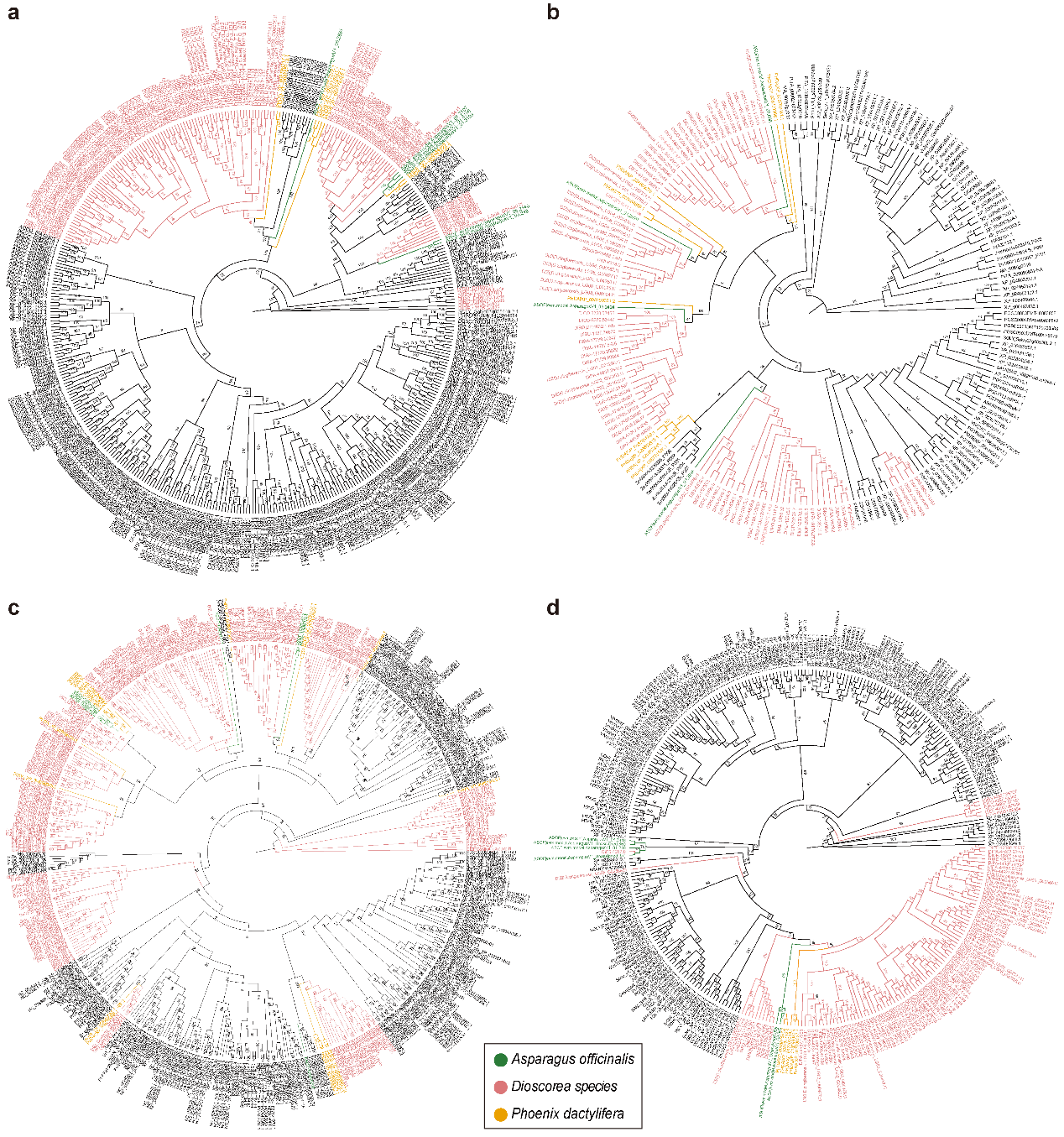
**

**Supplementary Fig. 15. Phylogenetic analysis of 4 key genes from different plants.** The tree is constructed from CYP72A (**a**), CYP90B (**b**), CYP94 (**c**), F26G (**d**) protein sequences of 12 *Dioscorea* plants and 32 selected plants (**Fig. 5a**) using RAxML with default parameters. The sequences from *A. officinalis*, *Dioscorea* species, and *P. dactylifera*, were marked in green, salmon, and orange, respectively.


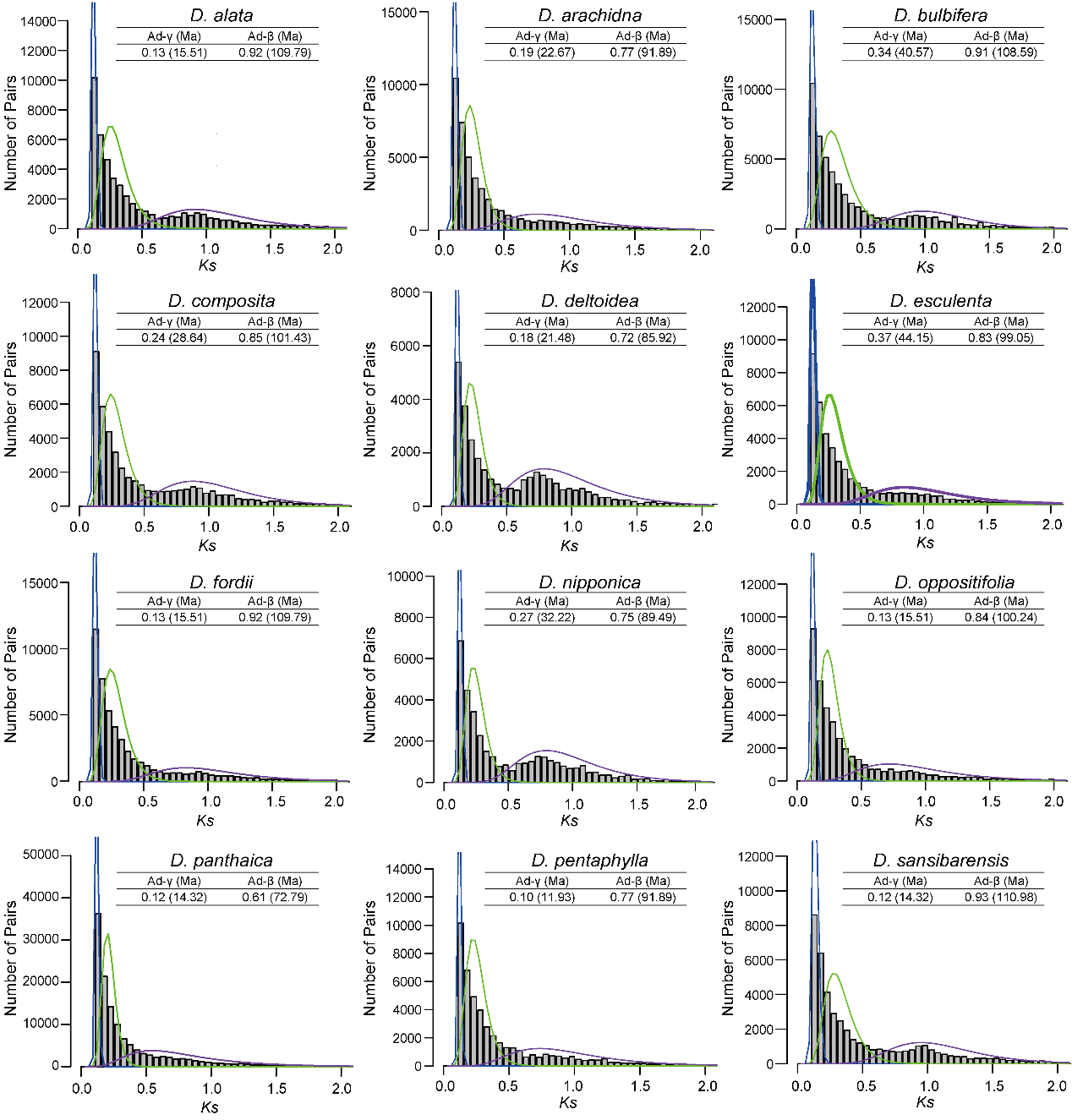


**Supplementary Fig. 16. *Ks* frequency plots of 12 *Dioscorea* species.** The *Ks* distribution frequency of each species was estimated and shown by mclust package. The alleles or sequencing errors were represented by the blue line; the background gene duplications were represented by green (Ad-α); and the putative WGD was represented by purple (Ad-β). The peaks of *Ks* value and the time of WGD events of each species were shown in the corresponding table.
